# Analysis of Short-Term Outcomes in Pancreatic Surgery with Vascular Resection from a Prospective Multicenter Global Study

**DOI:** 10.1245/s10434-025-17911-8

**Published:** 2025-08-26

**Authors:** Pascale Tinguely, Camila Hidalgo Salinas, Sebastian M. Staubli, Dimitri A. Raptis, Giuseppe K. Fusai, The Pancreasgroup.org Chief Investigator, Cristina Ferrone, The Pancreasgroup.org Scientific Committee collaborators, Mohamed Abu Hilal, Claudio Bassi, Marc Besselink, Kevin Conlon, Brian Davidson, Marco Del Chiaro, Christos Dervenis, Massimo Falconi, Thilo Hackert, Ewen Harrison, Shailesh V. Shrikhande, Ajith Siriwardena, Martin Smith, Christopher Wolfgang, The Pancreasgroup.org Management Committee collaborators, Aditya Borakati, Deniz Balci, Nikolaos Machairas, Giovanni Marchegiani, Atsushi Oba, Christian Oberkofler, Ioannis Passas, Reena Ravikumar, Patricia Sánchez Velázquez, Martin de Santibañes, Andreas Anton Schnitzbauer, Fiammetta Soggiu, Domenico Tamburrino, Alice Wei, Marinos Zachiotis, The Pancreasgroup.org Country Leaders, Kamel Bentabak, Salah Eddine Kacimi, Mehrdad Nikfarjam, Aliaksei Shcherba, Gregory Sergeant, Gustavo Coelho, Orlando Torres, Nikolay Belev, Burundi Fabrice, Ephraim Tang, Janet Martin, Christian Diaz, Nicolas Devaud, Kongyuan Wei, Maher Hendi, Danko Mikulic, Nikolaos Gouvas, Andrej Nikov, Dalia Fathallah, Mahmoud Saad, Olav Tammik, Heikki Huhta, Laurent Sulpice, Renato Lupinacci, Gregor Stavrou, Evangelos Felekouras, Vasileios Papaziogas, Sanjeev Misra, Erik Prabowo, Hashim Talib Hashim, Maytham Al-Juaifari Al-Sader, Sohei Satoi, Khaled Obeidat, Maram Mohsen, Ho-Seong Han, Mohamad Khalife, Muhammed Elhadi, Audrius Dulskas, Jin Bong, Shahi Ghani, Alejandro Eduardo Padilla, Javier Melchor-Ruan, Sarnai Erdene, Amine Benkabbou, Pueya Nashidengo, Jonathan Koea, Ademola Adeyeye, Olusegun Alatise, Sami Ullah, Mustafa Abu Jayyab, Sarah Amro, Walaa Mohammed Alnammourah, Catherine The, Michał Pędziwiatr, Wojciech Polkowski, Sorin Barbu, Aleksandar Karamarkovic, Daniel Galun, Brian Goh, Blaž Trotovšek, Jones Omoshoro-Jones, Benedetto Ielpo, Abdelfatah Abdelmageed, Per Sandström, Alessandra Cristaudi, Beat Gloor, Christoph Kuemmerli, Alaa Hamdan Tishreen, Mohammad Karam Chaaban, Chien Hui Wu, Po-Chih Yang Fu Jen, Ammar Houssem, Oussama Baraket, Ahmet Çoker, Mark Taylor, Nigel Jamieson, Satheesh Iype, Emmanouil Giorgakis, Motaz Qadan, Sabha Ganai, Hamza Al-Naggar, Onesai Chihaka, all Pancreasgroup.org collaborators, Amor El Behi, Anisse Tidjane, Ilhem Ouahab, Souad Bouaoud, Mounira Rais, Meriem Abdoun, Amel Ouyahia, Lucas Mc Cormack, Martin de Santibanes, Pablo Barros Schelotto, Shantanu Joglekar, Sivakumar Gananadha, Kat Hall, Russell Hodgson, Alistair Rowcroft, Lynn Chong, Kalpesh Shah, Stanley Chen, David Burnett, Zi Qin Ng, Christos Apostolou, Peter Kornprat, Radoslava Stoyanova, Aliaksei Shcherba, Bert Van den Bossche, Vera Hartman, Filip Gryspeerdt, Frederik Berrevoet, Sébastien Strypstein, Rinaldo Pinto, Gustavo Coelho, Matheus Militz, Pablo Rodrigues, Orlando Torres, Narimã Marques, Adriano Sampaio, Panche Krastev, Mihail Slavchev, Ivelin Takorov, Nikola Vladov, Vassil Mihaylov, Evgeni Nikolaev, Daniel Kostov, Pablo Serrano, Cristian Diaz, Alejandro Brañes, Lei Cai, Yiming Chen, Ivan Štironja, Tomislav Bubalo, Martin Loveček, Pavel Zaruba, Andrej Nikov, Almoatazbellah Attalla, Mosaab Tayiawi, Muneera Alboridy, Mohamed Mourad, Mostafa Elkeleny, Yousef Tanas, Hajer Ealreibi, Mohamed Abd El Moneam, Mohammed Hamouda, Ahmed Nafea, Mohamed Abdelalemm, Ahmed Mansour, Marina Farag, Mohamed Salah, Mohamed Abdelkarem, Hatem Sayed, Sherein Diab, Mira Gobran, Abdelrahman Fahim, Ahmed K. Awad, Sherif Abdelmawgoud, Emad Alazab, Mostafa Nagy, Islam Metwally, Ahmed Shehta, Ahmed Monier, Selmy Awad, Mohammed Omar, Feras Alsabbagh, Joonas Kauppila, Minna Nortunen, Heikki Huhta, Raffaele Brustia, Stéphanie Truant, Guillaume Piessen, Aurélien Dupré, Francois-Regis Souche, Natalia Savala, Renato Lupinacci, Sébastien Gaujoux, Nicolas Goasguen, Morgan Anyla, Lilian Schwarz, Fabio Giannone, Zaza Demetrashvili, Isabel Bartella, Ioannis Pozios, Orlin Belyaev, Dirk Bulian, Christian Praetorius, Sandra Korn, Maximilian Brunner, Elena Mazzella, Martin Reichert, Jochen Gaedcke, Ulrich Ronellenfitsch, Tim Reese, Kim Wagner, Pasquale Scognamiglio, Faik Uzunoglu, Jakob Izbicki, Benjamin Struecker, Dimitrios Kardassis, Silvio Nadalin, Georgios Makridis, Nikolaos Tsoukalas, Aristotelis Kechagias, Argyrios Ioannidis, Nikolaos Arkadopoulos, Dimitrios Papakonstantinou, Nikolaos Michalopoulos, Panagiotis Kokoropoulos, Pantelis Vassiliu, Dimitrios Stergiou, Maria Sotiropoulou, Nefeli Tomara, Panagiotis Dorovinis, George Tzimas, Dimitrios Schizas, Alexandros Papalampros, Andreas Polydorou, Georgios Fragulidis, Konstantinos Toutouzas, Antonios Vezakis, Konstantinos Bramis, Dimitrios Korkolis, Evangelos Fradelos, Georgios Glantzounis, Dimitrios Magouliotis, Grigorios Christodoulidis, Francesk Mulita, Elissaios Kontis, Vasileios Papaziogas, Dimitris Giakoustidis, Anastasios Katsourakis, Achilleas Νtinas, Panagiotis Petras, Attila Bursics, Andras Vereczkei, Sreenivasan Karuparthi, Govind Purushothaman, Rahul Gupta, Kaushal Yadav, Sundeep Jain, Jeewan Ram Vishnoi, Sanjeev Misra, Vaibhav Varshney, Kshaunish Das, Somnath Chattopadhyay, Jayapala Reddy Velagala, Varun Bansal, Induchoodan S, Tarun Kumar, Erik Prabowo, Hashim Talib Hashim, Maytham Al-Juaifari, Eran Sadot, Marco Vivarelli, Valeria Andriola, Michele Ciola, Mario Virgilio Papa, Adelmo Antonucci, Diego Sasia, Valentina Testa, Lapo Bencini, Tommaso Nelli, Fabrizio D’Acapito, Carlo Alberto Pacilio, Giacomo Carganico, Raffaele De Rosa, Stefano D’Ugo, Edoardo Saladino, Alfonso Recordare, Michele Mazzola, Vincenzo Mazzaferro, Alessandro Zerbi, Domenico Tamburrino, Caterina Baldi, Marco Stella, Fabrizio Di Benedetto, Fabio Uggeri, Alessandro Iacomino, Gianluca Rompianesi, Andrea Belli, Lucia Moletta, Francesca Tolin, Mario Giuffrida, Lorenzo Cobianchi, Paolo Regi, Luca Morelli, Emanuele Federico Kauffmann, Enrico Pinotti, Felice Giuliante, Alessandro Coppola, Tommaso Maria Manzia, Alberto Porcu, Claudio Feo, Teresa Perra, Francesco Ciarleglio, Simone Novello, Alessandro Ferrero, Sergio Intini, Giorgio Querini, Andrea Ruzzenente, Tommaso Campagnaro, Simone Conci, Sara Napetti, Alice Frontali, Daisuke Hashimoto, Ippei Matsumoto, Hiromitsu Maehira, Minoru Tanabe, Subhi Alissawi, Aiman Obed, Hebah Rababa, Khayry Al-Shami, Mohamed Alsabah, Saeed Shumrakh, Abdulaziz Al-Samawi, Almu’Atasim Khamees, Ildar Fakhradiyev, Ho-Seong Han, Kristaps Atstupens, Walid Faraj, Mohamad Khalife, Fatoom Alowjali, Abdulhadi Alshatshat, Aihab Benamwor, Eman Younes, Dania Burgan, Marwa Morgom, Muhannud Binnawara, Entisar Alshareea, Sultan Ahmeed Ahmeed, Eman Othman, Ahmed Gerwash, Osama Salem, Wegdan Khalil, Eman Abdulwahed, Mohamed Alharari, Tomas Vanagas, Vitalijus Eismontas, Jonas Jurgaitis, Algirdas Slepavicius, Vytenis Mikutaitis, Mindaugas Kvietkauskas, Edoardo Rosso, Andee Dzulkarnaen Zakaria, Ian Chik, Jin Bong, Carlos Florez Zorrilla, Sarnai Erdene, Moniba Korch, Iltimass Gouazar, Badr Serji, Aziz Zentar, Amine Benkabbou, Reda Elhassouni, Sabrillah Echiguer, Mohammed Anass Majbar, Abdulrashid Pueya Nashidengo, Paleswan Joshi Lakhey, John Windsor, Michael Jen Jie Chu, Peter Johnston, Vanshay Bindra, Andrea Cross, Ashok Gunawardene, Fraser Welsh, Olusegun Alatise, Jibran Abbasy, Tayyab Siddiqui, Mohammad Asghar, Mustafa Abu Jayyab, Qusai Zreqat, Rawand Titi, Fatima Manasrah, Ahlam Hammoudeh, Guillermo Coayla, Catherine Teh, Cenon Alfonso, Marta Flisińska, Justyna Rymarowicz, Marek Sierzega, Wojciech Ciesielski, Oliwia Grząsiak, Patrycja Szewczyk, Krzysztof Szwedziak, Wojciech Korcz, Oskar Kornasiewicz, Emanuel Vigia, Raluca Bievel Radulescu, Traian Dumitrascu, Mara Mardare, Octav Ginghina, Iulian Brezean, Sorin Petrea, Adrian Bartos, Raluca Bodea, Sergiu Matei, Ana-Maria Musina, Natalia Velenciuc, Alexander Belyaev, Denis Mizgirev, Andrey Litvin, Ivan Semenenko, Arkady Bedzhanyan, Nikolay Bagmet, Ayrat Kaldarov, Denis Kuchin, Evgeniy Drozdov, Mahir Gachabayov, Mohammed Alharthi, Aleksandar Bogdanovic, Daniel Galun, Stefan Kmezic, Dragana Arbutina, Mihailo Bezmarevic, Jovan Juloski, Mladjan Protic, Brian Goh, Blaž Trotovšek, Emil Loots, Jones Omoshoro-Jones, Manuel Marcello, Jose Ramia, Maria Del Mar Rico-Morales, Fabio Ausania, Ana Belen Martin Arnau, Benedetto Ielpo, Esther Pilar Santos, Patricia Ruiz, Laia Falgueras, Farah Al Shwely, Juli Busquets, Iago Justo, Jana Dziakova, Marcello Di Martino, Miguel Ángel Suárez-Muñoz, Lorena Solar García, Juan Jose Segura-Sampedro, Fernando Rotellar, Luis Muñoz-Bellvis, Valle Vera, María García Domingo, Carlos Domingo-Del Pozo, Cristina Ballester Ibáñez, Dimitri Dorcaratto, Mario Rodriguez-Lopez, Alejandro Serrablo, Hytham Hamid, Abdelfatah Abdelmageed, Per Sandström, Linda Lundgren, Bobby Tingstedt, Caroline Williamsson, Bodil Andersson, Ernesto Sparrelid, Christoph Kuemmerli, Beat Moeckli, Alessandra Cristaudi, Fariba Abbassi, Jan Schmidt, Stefan Gutknecht, Jan Philipp Jonas, Mais Alhashemi, Zain Douba, Ahmad Kayali, Oula Azizeh, Omar Al-Abed, Aya Abdulmonem, Ahmad Alhouri, Hasan Al Houri, Alnour Suliman, Bashar Haj Hassan, Alaa Hamdan, Ming-Chin Yu, Hanen Bouaziz, Ali Kchaou, Ammar Houssem, Mesut Tez, Mustafa Kerem, Hüseyin Bayhan, Ulaş Aday, Alpen Gumusoglu, Husnu Aydin, Fatema Hanefa, Ali Emre Atici, Hanife Ulgur, Ismail Sert, Semra Demirli Atici, Elif Colak, Denys Skoryi, Kostiantyn Kopchak, Oleksandr Kvasivka, Valeriia Sumarokova, James Skipworth, William Lim, Ahmed Mohamed, Pascale Tinguely, Hemant Kocher, Amjad Khalil, Nicola de’ Liguori Carino, Fahed Gareb, Pandanaboyana Sanjay, Krishnakumure Patel, Carlo Ceresa, Thomas Russell, Matt Mortimer, Megan Sulciner, Thomas Clancy, Martina Nebbia, Rachel Thompson, Dimitrios Moris, Jennifer Gnerlich, Richard Spencer-Cole, Derek Krinock, Tamara Osborn, Hailey Hardgrave, Joe Nigh, Beth Schrope, Ryan Lamm, Harish Lavu, Wilbur Bowne, Jonathan Sham, Paulo Martins, Aisha Albar, Fatima Al-Eryani, Hamza Al-Naggar, Rafat Al-Saban

**Affiliations:** 1 Department Of HPB Surgery and Liver Transplant, Royal Free Hospital NHS Foundation Trust, London, UK; 2 Centre For Evidence-Based Medicine, Nuffield Department of Primary Care Health Sciences, University of Oxford, Radcliffe Observatory Quarter, Oxford, UK; 3 Department For Continuing Education, University of Oxford, Oxford, UK; 4 King Faisal Specialist Hospital & Research Centre, Organ Transplant Centre of Excellence, Riyadh, Saudi Arabia; 5 Department of Surgical Biotechnology, University College London, London, UK

**Keywords:** Pancreas surgery, Mortality, Morbidity, Vascular resection, Pancreatic neoplasms, Pancreatoduodenectomy

## Abstract

**Background:**

Pancreatic resection with concomitant vascular resection is increasingly practiced with outcomes mainly reported from specialist centers but lacking results from prospective global data. This study aimed to investigate factors associated with short-term outcomes after vascular resections in pancreatic surgery worldwide.

**Patients and Methods:**

Data were extracted from a prospective, multicenter, international cross-sectional snapshot study in 2021 (pancreasgroup.org) assessing short-term outcomes after pancreatic surgery worldwide (NCT04652271). In the patient cohort of pancreatic surgery with simultaneous vascular resection for various diseases, short-term outcomes were reported and compared with established benchmark values. Factors affecting major complications, mortality, and histopathological resection status were assessed in multivariable logistic regression analyses with interaction testing.

**Results:**

From a total of 3926 patients undergoing pancreatic surgery, 565 had associated vascular resections, of which 444 had venous resections alone and 121 had arterial resections alone or with concomitant venous resection. Of the 153 (47%) benchmark cases with pancreatoduodenectomy and venous resection, median postoperative morbidity fell within established benchmark criteria. Median 90-day major complication and mortality rates were similar in pancreatic resection with venous, arterial or no vascular resections (45 and 10%, 47 and 6.6%, 42 and 9.6% respectively). Patients undergoing arterial resections that developed a clinically relevant pancreatic fistula faced substantially elevated odds of 90-day mortality (OR 8.8 CI 1.6–48). In pancreatic ductal adenocarcinoma, the R1 rate was 26%, neoadjuvant chemotherapy being protective for both overall and venous-specific margins.

**Conclusions:**

Vascular pancreatic surgery is performed across diverse healthcare settings worldwide. While perioperative complications were comparable to nonvascular pancreatic resections, the observed 90-day mortality was considerable overall. International collaborative efforts should focus on understanding practice variations and improve accessibility of optimal perioperative care to promote rescue capabilities.

**Supplementary Information:**

The online version contains supplementary material available at 10.1245/s10434-025-17911-8.

Surgical resection remains the only potentially curative option in malignant disease originating from the pancreas or distal bile duct, and occasionally is necessary for benign and premalignant conditions as well.^1–3^ The presence of vascular involvement significantly increases the degree of surgical complexity in patients requiring pancreatic surgery, including simultaneous portomesenteric venous and arterial resections. The favorable safety, efficacy, and oncological outcomes reported from specialized pancreatic centers has encouraged surgeons worldwide to adopt this practice.^4–6^ In pancreatic ductal adenocarcinoma (PDAC) specifically, a shift in paradigm regarding resection as a continuum of oncological care was further introduced by neoadjuvant treatments, depending on factors such as tumor localization, type of affected vasculature, and degree of vascular involvement.^7^

Nevertheless, several controversies remain in the clinical decision-making and treatment evaluation for patients needing combined pancreatic and vascular resections globally. The generalization of favorable results, mainly reported from specialized high-volume centers, to a global surgical community, including centers from lower resource environments, is difficult, considering the differences in short-term outcomes reported across countries of varying Human Development Index (HDI) and associated learning curves.^5,8^ True outcomes and current practice of such highly complex procedures from worldwide cohorts remain mostly unknown. In addition, international reports on the effect of neoadjuvant treatment on short-term outcomes and specific resection margins in pancreatic with simultaneous vascular resections remain scarce.^9–11^

The aim of this study was to assess factors associated with perioperative morbidity and histological resection margins, including the role of neoadjuvant treatment, in a worldwide prospective cohort of pancreatic surgery with concomitant vascular resection.

## Patients and Methods

### Data and Patient Cohort

Data from a prospective, multicenter, cross-sectional snapshot study of patients undergoing pancreatic surgery across the globe in 2021 were used in this study (pancreasgroup.org, NCT04652271, ISRCTN95140761).^8^ The pancreasgroup.org study was a prospective study of consecutive patients undergoing pancreatic resections over a predefined period of 3 months in 2021 with a 90-day follow-up. Routine secondary data were collected and no changes to clinical care were made. The principal investigator at each participating center was responsible for their appropriate institutional research committee compliance, according to local and national regulations. We abided by the STrengthening the Reporting of OBservational studies in Epidemiology (STROBE) guidelines.^12^

### Inclusion and Exclusion Criteria

In this study, only adult patients undergoing pancreatic resections were included, irrespective of indication (benign or malignant), operative approach (open, laparoscopic, or robotic), timing (elective or emergency), extent (partial or total pancreatectomy), vascular resection and type thereof, and concomitant resection of other organs. Pancreatic biopsies, abandoned pancreatic resections, bypass procedures, drainage procedures, pancreas transplantation (whole organ or islet cell), and ablation or necrosectomy procedures were excluded. There were no restrictions on hospital or procedural volumes for participating centers. This was deliberately set to obtain a representative global sample and allow for the inclusion of cases from surgical centers in countries with limited resources, where case volumes are typically low.

### Outcomes and Definitions

Postoperative complications were documented according to the Clavien–Dindo classification of surgical complication.^13^ Clavien–Dindo grade ≥ 3a complications were considered major complications.^13,14^ The comprehensive complication index^®^ (CCI)^15,16^ at discharge was calculated retrospectively. Failure-to-rescue (FTR) was calculated as the number of patients that died over the total number of patients with major postoperative complications on postoperative day 90.^17^ The HDI categories were based on the relevant data for the year 2021.^18^ Benchmark patients undergoing pancreatoduodenectomy with simultaneous venous resection were selected according to the established benchmark criteria, which included body mass index (BMI) < 35, American Society of Anesthesiologists (ASA) < 3, no severe cardiac disease, no severe chronic obstructive pulmonary disease, no insulin requirement, no arterial resection, and no minimally invasive surgery.^4^

### Statistical Methods

We report baseline and perioperative characteristics as well as postoperative outcomes as median and interquartile range or as frequency and percentage, as appropriate. Descriptive outcome data across groups was compared using Fisher’s exact for binary or categorical data and the Kruskal–Wallis test for continuous data. Benchmark criteria were used to compare outcomes in patients undergoing venous resections. Exploratory multivariable logistic regression was performed to investigate the association of baseline and perioperative factors with (i) major complication rate overall and in the specific subgroup of patients undergoing pancreatoduodenectomy and (ii) histopathological resection margin status (R0 versus R1/R2, overall and for venous margins specifically) in PDAC. Baseline characteristics available in our patient cohort known or thought to affect these outcomes were included in the multivariable analyses. Exploratory interaction, in limited unadjusted logistic regression, between clinically relevant complications and types of vascular resection on mortality, was conducted prior to multivariable logistic regression on mortality. Multivariable analysis was also performed to assess factors potentially associated with 90-day mortality introducing interaction terms significant in exploratory analysis.

Effect sizes and ranges of uncertainty were reported as odds ratios (OR) and 95% confidence intervals (CI). Basic assumptions justifying logistic regression analysis (multicollinearity of independent variables, linearity between independent variables, and dependent variables’ log odds) were analyzed and found as satisfied. The threshold for statistical significance was set to alpha < 0.05.

### Missing Data

We excluded covariates with over 20% missing observations from regression analyses. We assessed the nature of missingness in covariates missing less than 20% of data and used multiple imputation by chained equations to impute age and pancreatic gland texture, which were deemed missing at random (MAR). The potential explanations for missingness of imputed variables did not appear to correlate with the clinical outcome. We performed sensitivity analysis of imputed against complete case in multivariable regressions to assess any departures from the MAR assumption. The imputation model, with 40 imputed datasets, included the selected regression covariates, outcome variable, final histopathology, preoperative radiation therapy, HDI, and preoperative comorbidities.

We performed all statistical analyses using STATA/IC version 18.0 (STATACORP, 4905 Lakeway Dr College Station, TX 77845, USA).

## Results

### Cohort Characteristics

**Fig 1:**
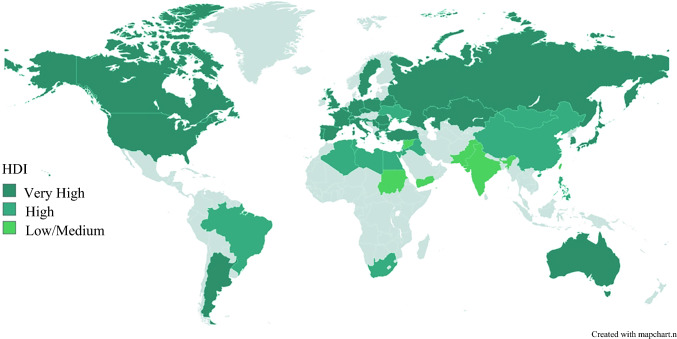
World map of contributing countries according to the 2020 Human Development Index

From the pancreasgroup.org cohort comprising 4223 patients across 317 centers worldwide, 3925 met the inclusion criteria for this study, of whom 565 (14%) patients underwent vascular pancreatic surgery from 167 centers. Among the patients undergoing vascular resections, 444 (79%) had venous resections and 121 (21%) had arterial resections with or without concomitant venous resection. Vascular pancreatic surgery was performed in 52 countries and from Asia, Africa, North America, South America, Europe, and Oceania, of which 458, 91, and 16 patients were treated in very high, high, and medium/low HDI countries, respectively (Fig. 1). The main indication for vascular pancreatic surgery was PDAC in 429 (76%) patients, and the most commonly performed pancreatic resections were pancreatoduodenectomy, total pancreatectomy, and distal pancreatectomy in 396 (70%), 91 (16%), and 77 (14%) patients, respectively (Table 1). For PDAC, neoadjuvant chemotherapy was administered in 434 (23%) patients overall and in 156 (36%) undergoing vascular pancreatic surgery, with 54% of patients undergoing arterial resection receiving neoadjuvant/induction chemotherapy.

**Table 1:** Baseline and perioperative characteristics of patients undergoing pancreatic resections

*N* 3926	Without vascular resection *n* = 3360	With concomitant venous resection only *n* = 444	With concomitant arterial resection ± venous resection *n* = 121
Patient demographics			
Age	65 (55–72)	67 (59–73)	66 (56–73)
Age > 65	1583 (47)	239 (54)	63 (52)
Missing data	3 (0.1)	1 (0.2)	–
Male sex	1,741(52)	208 (47)	67 (55)
BMI	24.9 (22.2–27.8)	24.3 (22.0–27.0)	23.7 (21.3–27.1)
BMI > 35	110 (3.3)	12 (2.7)	2 (1.7)
Missing data	43 (1.3)	6 (1.4)	–
ASA > 2	1119 (33)	168 (38)	56 (46)
Charlson comorbidity index > 2	1115 (33)	187 (42)	53 (44)
Charlson comorbidity index > 4	196 (6)	33 (7)	14 (12)
Country HDI group			
Very high	2803 (84)	380 (86)	78 (64)
High	345 (10)	59 (13)	32 (26)
Mid/low	212 (6)	5 (1)	11 (9)
Preoperative therapy			
Biliary drainage	1109 (33)	186 (42)	46 (38)
Missing data	20 (0.6)	4 (0.9)	–
Preop chemotherapy alone	326 (10)	118 (27)	43 (36)
FOLFIRINOX	166 (51)	73 (62)	29 (67)
Gemcitabine-abraxane	43 (13)	28 (24)	9 (21)
Other	113 (35)	17 (14)	5 (12)
Missing data	3 (0.9)	–	–
Preop chemoradiation	53 (2)	25 (6)	10 (8)
Preop radiotherapy alone	6 (0.2)	2 (0.5)	2 (1.7)
Type of pancreatic resection			
Pancreatoduodenectomy	2153 (64)	328 (74)	68 (56)
Total pancreatectomy	208 (6)	66 (15)	25 (21)
Distal pancreatectomy	978 (29)	49 (11)	28 (23)
Central pancreatectomy	21 (0.6)	1 (0.2)	–
Surgical approach			
Open surgery	2864 (85)	434 (98)	118 (98)
Minimally invasive	496 (15)	10 (2)	3 (2)
Type of vascular reconstruction			
Tangential	N/A	170 (38)	14 (12)
End-to-end	N/A	197 (44)	34 (28)
Autologous or cadaveric graft	N/A	33 (7)	7 (6)
Prosthetic graft	N/A	26 (6)	1 (1)
Missing data	N/A	18 (4)	65 (54)
Gland characteristics			
Soft	1792 (53)	178 (40)	35 (29)
Missing data	482 (14)	54 (12)	29 (24)
Duct size smaller than 3 mm	998 (30)	116 (26)	28 (23)
Missing data	822 (24)	77 (17)	45 (37)
Intraoperative therapy			
Intraoperative blood transfusion	690 (21)	141 (32)	55 (45)
Intra- and/or postop octreotide	1216 (36)	143 (32)	50 (41)
Pathology			
PDAC	1423 (42)	351 (79)	78 (64)
Cholangiocarcinoma	200 (6)	26 (6)	7 (6)
Neuroendocrine tumor	335 (10)	19 (4)	7 (6)
Ampullary carcinoma	388 (12)	13 (3)	12 (10)
Chronic pancreatitis	167 (5)	8 (2)	8 (7)
IPMN	235 (7)	7 (2)	2 (2)
Duodenal adenocarcinoma	108 (3)	6 (1)	2 (2)

Values are shown as medians and (IQR) or numbers and (percentages) as appropriate.

*HDI* Human Development Index, *BMI* body mass index, *ASA* American Society of Anesthesiologists, *COPD* chronic obstructive pulmonary disease, *PDAC* pancreatic ductal adenocarcinoma, *IPMN* intraductal papillary mucinous neoplasm, *PPPD* pylorus-preserving pancreatico-duodenectomy, *PD* pancreatico-duodenectomy

### Short-Term Clinical Outcomes

A complication of any grade was observed in 3513 (90%) patients overall within 90 days postoperatively, with comparable rates regardless of whether vascular resection was performed or type of vascular resection (*p* = 0.553) (Table 2). The median CCI score until hospital discharge overall was 8.7 (IQR 0–30). Median CCI was higher in patients undergoing vascular resection (*p* < 0.001) and it was higher in patients undergoing arterial resection than in venous resection only (*p* = 0.011). A total of 1664 (42%) patients experienced a major complication overall. Among patients undergoing arterial resections, 57 (47%) experienced a major complication; however, no statistical difference was observed across patients without vascular resection or venous or arterial resection in our cohort (*p* = 0.284). The most common clinically relevant complications directly related to the pancreatic procedure were pancreatic fistula (14%, 500/3639), delayed gastric emptying (8%, 299/3603), postoperative hemorrhage (7%, 252/3586), and biliary leakage (3%, 115/3585), with 4% (99/2329) in the pancreatoduodenectomy subgroup and none in the total pancreatectomy subgroup. In patients undergoing venous resections, with or without arterial resection, 4% (20/500) developed portal vein thrombosis, among which 11 patients required no treatment or only pharmacological treatment, while 2 died as a result. The venous thrombosis rate was significantly lower in the direct (tangential or end-to-end) than in the graft (autologous, cadaveric, or prosthetic) reconstruction (3% [11/411] versus 12% [8/67] respectively, *p* = 0.002).

**Table 2:** Postoperative outcomes of patients undergoing pancreatic resections

	Without vascular resection *n* = 3360	With concomitant venous resection only *n* = 444	With concomitant arterial resection ± venous resection *n* = 121
Complications			
Any complication (90 days)	3013 (90)	391 (88)	109 (90)
CCI (at discharge)	8.7 (0–30)	21 (0–31)	23 (0–37)
Major complications (90 days)	1408 (42)	199 (45)	57 (47)
POPF B/C	459 (14)	30 (7)	11 (9)
Missing	239 (7)	23 (5)	24 (20)
DGE B/C	244 (7)	40 (9)	15 (12)
Missing	270 (8)	26 (6)	26 (21)
PPH B/C	252 (6)	28 (6)	9 (7)
Missing	339 (9)	27 (6)	25 (21)
Bile leak B/C	94 (3)	17 (4)	4 (3)
Missing	290 (9)	25 (6)	25 (21)
Mortality (90 days)	323 (10)	46 (10)	8 (7)
Failure-to-rescue (FTR) rate	23% (322/1,408)	23% (46/199)	14% (8/57)
Length of stay			
Intensive/high/intermediate dependency unit stay	1 (0–3)	2 (1–5)	3 (1–7)
Length of hospital stay	13 (7–17)	13 (9–20)	12 (7–19)
Readmission within 90 days	574 (17)	67 (15)	18 (15)
Missing data	434 (13)	61 (14)	12 (10)
Histology for PDAC only (*n* = 1852)			
Lymphovascular invasion	804 (57)	231 (66)	42 (54)
Perineural invasion	985 (69)	283 (81)	47 (60)
R1 margin	321 (23)	103 (29)	22 (28)
Posterior margin	153 (11)	44 (13)	16 (21)
SMV margin	84 (6)	34 (10)	6 (8)
Portal vein margin	4 (0.3)	14 (4)	2 (3)
SMA margin	62 (4)	23 (7)	7 (9)
Pancreas transection margin	49 (4)	23 (7)	9 (12)
Bile duct margin	13 (1)	–	6 (8)
Gastric/proximal jejunum	5 (0.4)	1 (0.3)	8 (10)
Distal duodenum	5 (0.4)	–	5 (6)
Other	16 (1)	2 (1)	1 (1)

Values are shown as numbers and (percentages).

*CCI* comprehensive complication index, *POPF* postoperative pancreatic fistula, *DGE* delayed gastric emptying, *PPH* postoperative hemorrhage, *SMV* superior mesenteric vein, *SMA* superior mesenteric artery

**Fig. 2:**
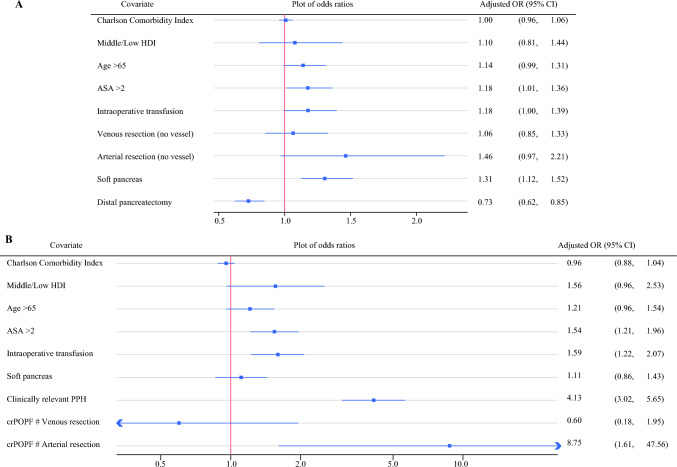
Forest plots of multivariable analyses of **A** 90-day major morbidity in the overall cohort (no vessel resection as reference category for venous and arterial resections) and **B** 90-day mortality with an interaction term for clinically relevant postoperative pancreatic fistula (crPOPF) and vascular resection (full factorial interactions with main effects factored into the effect estimates,  however not displayed in forest plot for clarity)

On multivariable analysis following multiple imputation, soft pancreatic texture, ASA over two, and intraoperative transfusion emerged as independent predictors of 90-day major complication rate. Although not statistically significant (*p* = 0.070), the point estimate of the odds ratio for arterial resection and reconstruction (OR 1.46, 95% CI 0.97–2.21) was higher than for venous resection alone (OR 1.06, 95% CI 0.85–1.33; no vascular resection as reference category (Fig. 2A).

**Fig. 3:**
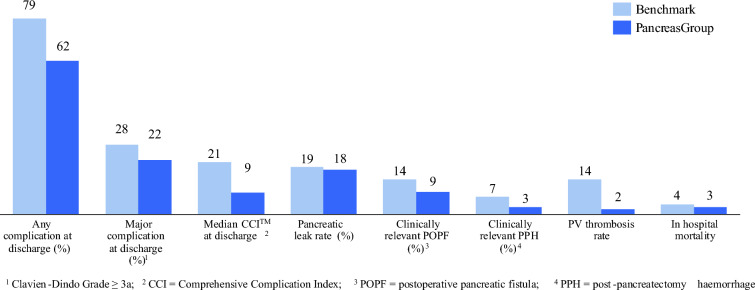
Bar graph of postoperative complications at discharge in benchmark cohort undergoing pancreatoduodenectomy with simultaneous venous resection

In the subgroup of patients undergoing pancreatoduodenectomy with venous resection, 47% of cases qualified as benchmark cases. Across HDI groups, the rates of benchmark cases were lowest in very high HDI (43%), while in countries of high and low/medium HDI they were 65% and 67%, respectively. All postoperative morbidity benchmark outcomes at discharge captured in this cohort lied within established benchmark limits (Fig. 3).

Postoperative mortality within 90 days was 9.6% (377/3,925), with similar rates between arterial and venous resection groups (*p* = 0.294) (Table 2). We identified a significantly relevant interaction between clinically relevant postoperative pancreatic fistula (POPF) and arterial resection (OR 6.2 [95% CI 1.2–31.1]) on exploratory interaction analyses. On multivariable analysis, while arterial resection alone (OR 0.4 [95% CI 0.14–1.13]) and clinically relevant POPF (OR 1.5 [95% CI 1.1–2.0]) did not increase mortality risk, patients that developed a clinically relevant POPF having undergone arterial resection (OR 8.8 [95% CI 1.6–47.6]) faced substantially elevated mortality risk even after controlling for age, ASA, and Charlson comorbidity index (Fig. 2B). No significant interactions were observed between vascular resection type and clinically relevant delayed gastric emptying (DGE), bile leak, or postoperative hemorrhage (PPH). However, clinically relevant PPH demonstrated a consistently strong association with 90-day mortality regardless of vascular resection type (OR 4.1 [95% CI 3.0–5.6]) in multivariable analysis. Other covariates significantly associated with 90-day mortality in this analysis included ASA over two and intraoperative transfusion.

The overall failure-to-rescue (FTR) rate was 23%, which remained consistent among patients undergoing venous-only resections (23%). In contrast, patients who underwent arterial resections demonstrated an FTR rate of 14%. Multivariable logistic regression controlling for ASA grade, Charlson comorbidity index, and age showed no significant association between vascular resection type and FTR (arterial OR 0.53 [95% CI 0.26–1.06]). Notably, patients undergoing arterial resections had a significantly higher prevalence of ASA sores > 2 compared with venous resections (*p* = 0.003).

### Histopathological Margin Status

The overall positive resection margin rate in patients undergoing vascular resection was 29%, with the most frequently affected margin being the posterior margin in 13%. In patients with PDAC, the positive resection margin rate was 26%, the most frequent R1 site being the posterior margin in 12% (Table 2). In these patients, multivariable analyses of the overall resection margin status showed that the type of vascular resection (arterial versus nonarterial) did not affect R1 status (OR 1.57, CI 0.89–2.76, *p* = 0.119), while the administration of neoadjuvant chemotherapy was associated with a lower R1 (OR 0.61, CI 0.39–0.97, *p* = 0.036), adjusting for the tumor’s pathological T-stage and N-stage. Equally, in patients with PDAC undergoing venous resections, neoadjuvant chemotherapy was protective for a positive venous resection margin (superior mesenteric vein [SMV] and/or portal vein [PV] margin) (OR 0.48, CI 0.24–0.99, *p* = 0.049), adjusting for the tumor’s pathological T-stage and N-stage.

## Discussion

This study demonstrates that pancreatic surgery with vascular resection, including arterial resection and reconstruction, is performed globally with postoperative morbidity rates comparable to nonvascular resection. To our knowledge, this represents the first prospective report of outcomes after vascular pancreatic surgery in a global patient collective, including institutions of varying case volume from a range of healthcare systems, including medium and low HDI countries, providing real-world evidence. Critically, the major complication rates in patients from medium and low HDI were comparable to those from high and very high HDI settings, challenging assumptions that resource limitations necessarily translate to inferior surgical outcomes. However, the nonsignificant trend toward higher mortality in medium and low HDI countries suggests that while technical surgical execution remains consistent globally, differences in complication management and rescue capabilities may be the determining factors for ultimate patient outcomes. This finding underscores the importance of investigating failure-to-rescue mechanisms in vascular pancreatic surgery across different healthcare environments.

Overall in-hospital complications were comparable to previous reports and within established benchmark criteria in the subgroup of patients undergoing pancreatoduodenectomy and venous resection.^4^ However, our observed overall 90-day mortality of 9.6%—with similar rates between arterial and venous pancreatic surgery—exceeded previously reported rates of 6.3% and 5.1% in a recent large series and meta-analysis of pancreatic resections with venous reconstruction.^6,19^ Potential explanations for the elevated mortality in our cohort may be related to the methodology applied by the pancreasgroup.org study, which was aimed at the inclusion of cases specifically from centers where case volumes are typically lower, and reporting an unselected patient population. As a result, this introduces heterogeneity in practice that allows us to examine the true global practice of vascular pancreatic surgery, giving centers that are under reported in the literature a platform to report their outcomes. The significant interaction between arterial resection and clinically relevant POPF resulting in an eightfold increase in odds of mortality, for example, represents a crucial finding that may influence perioperative management strategies. To support this, we observe lower rates of pancreatoduodenectomy but higher rates of total pancreatectomy in patients undergoing arterial resection, suggesting a potential approach to mitigate mortality risk in the setting of POPF.

Examining mortality in relation to major complications, FTR rates were 14% in patients undergoing arterial resection compared with 23% in venous resection and nonresection patients. Importantly, patient selection factors including age and comorbidities did not explain the observed variation in FTR rates. Notably, patients undergoing arterial resection had a significantly higher rate of ASA scores > 2 than venous resections. Given our global patient cohort from very heterogeneous environments with varying expertise, resources, and case volume, this finding may thus reflect hospital-related factors influencing rescue capabilities, whereby centers with superior rescue infrastructure undertake more complex arterial resections in increasingly comorbid patients. While this warrants further investigation, the lower rates of benchmark patients in very high HDI compared with high and middle/low HDI countries may indicate that centers with potentially higher resources are able to undertake these operations in complex patients without increased risk in surgical outcomes. Factors beyond patient and disease-related aspects, such as case volume (not available in the current analysis) and resource-bound aspects on an institutional level, possibly affect FTR in pancreatic surgery,^20–22^ which our group is currently investigating across the worldwide pancreasgroup.org network.^23^

Another finding that underscores the real-world oncological practice patterns was the generally low rate of neoadjuvant chemotherapy (36% in the subgroup of all arterial resections). This is particularly notable given that current guidelines recommend neoadjuvant therapy for all borderline resectable and locally advanced disease^1^. Although we lacked data on initial resectability status, this observation highlights critical gaps between recommendations and clinical implementation on a global scale. These findings emphasize the need to further investigate real-world adherence to guidelines across diverse healthcare systems and underscores the importance of global health initiatives aimed at improving chemotherapy accessibility.

Surgical resection margin status is one of the prognostic factors affecting long-term survival after surgery for patients with PDAC.^6,7,25^ In the present global patient collective, R1 resection status was lower than reported in other reports on venous resections.^22^ A probable reason may be the lack of a universally accepted definition in resection margin status for PDAC,^26,27^ with “R1” generally defined as the presence of tumor cells < 1 mm from the resection margin as per the British Royal College Of Pathologists in Europe, and a zero millimeter rule (presence of residual cells at the cut surface) in North America, leading to a discrepancy in the histopathological reporting between different continents. Knowing the benefits of using standardized pathological protocols in predicting oncological outcomes,^28^ the importance of generalizable reporting of resection margins on a global level has been highlighted.^11,27^ Neoadjuvant chemotherapy led to lower R1 rates (OR 0.61) in our vascular surgery cohort and lower venous margin positivity (OR 0.48) in patients with PDAC, providing strong support for neoadjuvant treatment in borderline resectable and locally advanced disease, aligning with previous reports,^10,29–31^ as well as in the venous resection margin specifically. While most patients received a FOLFIRINOX regimen, no subgroup analysis on type of neoadjuvant therapy were made owing to small numbers in individual subgroups. Controversy exists whether the specific location of positive margins affect oncological outcomes,^32,33^ and future research on this topic, and specifically in vascular resections, might add substantial knowledge to the field.

The most important limitation of this study is the heterogeneity in data collection, arising from the very nature of this truly international worldwide pancreasgroup.org snapshot study, introducing inherent selection bias and potential variations in data quality and completeness. Maximum efforts to limit heterogeneity were made in the design of pancreasgroup.org study, providing definitions for individual variables and applying restrictions for multilayered variables, while multiple imputation was used in this analysis to address data incompleteness. However, differences in the understanding of individual parameters across countries and continents might have introduced a non-quantifiable bias in data assessment. The interpretation of R1 status must be considered within the broader context of its limitations as a surrogate endpoint in pancreatic cancer outcomes. R1 status is inherently dependent on pathologist expertise, introducing significant interobserver variability that may confound outcome comparisons across our global cohort. This pathologist-dependent variability is particularly relevant in our international study, where protocols vary considerably between healthcare systems. Furthermore, while R1 status serves as an important prognostic marker, it represents only one component of the complex oncological picture in PDAC, where factors such as tumor biology, response to systemic therapy, and overall disease burden often outweigh margin status in determining long-term survival. The relatively low R1 rates observed in our study, particularly in the context of complex vascular resections, should therefore be interpreted cautiously given these methodological limitations. Since the pancreasgroup.org study was designed for short-term outcomes after all pancreatic surgery, individual variables more specific to vascular resections, such as the response to neoadjuvant chemotherapy, the reason for vascular resection, and the center’s surgical volume/expertise, were not available in this cohort. Importantly, this included data on the initial resectability status—up-front versus borderline resectable versus locally advanced disease—and detailed radiological resectability criteria, which is lacking and therefore precluded conclusions specific to either subgroup.^10^

In conclusion, vascular pancreatic surgery demonstrates comparable complication rates globally across all HDI levels, with mortality differences reflecting rescue capabilities rather than surgical technique. The eightfold mortality increase from arterial resection-pancreatic fistula interaction and suboptimal neoadjuvant therapy adherence represent critical targets for improvement. Future research must investigate institutional rescue factors to optimize global outcomes.

## Supplementary Information

Below is the link to the electronic supplementary material.

Supplementary file1 (DOCX 75 KB)

## Data Availability

The dataset analyzed in this study is not publicly available but can be made available upon reasonable request to the corresponding author.
