# Correction: Analysis of Short-Term Outcomes in Pancreatic Surgery with Vascular Resection from a Prospective Multicenter Global Study

**DOI:** 10.1245/s10434-025-18348-9

**Published:** 2025-09-07

**Authors:** Pascale Tinguely, Camila Hidalgo Salinas, Sebastian M. Staubli, Dimitri A. Raptis, Giuseppe K. Fusai, The Pancreasgroup.org Chief Investigator, Cristina Ferrone, The Pancreasgroup.org Scientific Committee collaborators, Mohamed Abu Hilal, Claudio Bassi, Marc Besselink, Kevin Conlon, Brian Davidson, Marco Del Chiaro, Christos Dervenis, Massimo Falconi, Thilo Hackert, Ewen Harrison, Shailesh V. Shrikhande, Ajith Siriwardena, Martin Smith, Christopher Wolfgang, The Pancreasgroup.org Management Committee collaborators, Aditya Borakati, Deniz Balci, Nikolaos Machairas, Giovanni Marchegiani, Atsushi Oba, Christian Oberkofler, Ioannis Passas, Reena Ravikumar, Patricia Sánchez Velázquez, Martin de Santibañes, Andreas Anton Schnitzbauer, Fiammetta Soggiu, Domenico Tamburrino, Alice Wei, Marinos Zachiotis, The Pancreasgroup.org Country Leaders, Kamel Bentabak, Salah Eddine Kacimi, Mehrdad Nikfarjam, Aliaksei Shcherba, Gregory Sergeant, Gustavo Coelho, Orlando Torres, Nikolay Belev, Burundi Fabrice, Ephraim Tang, Janet Martin, Christian Diaz, Nicolas Devaud, Kongyuan Wei, Maher Hendi, Danko Mikulic, Nikolaos Gouvas, Andrej Nikov, Dalia Fathallah, Mahmoud Saad, Olav Tammik, Heikki Huhta, Laurent Sulpice, Renato Lupinacci, Gregor Stavrou, Evangelos Felekouras, Vasileios Papaziogas, Sanjeev Misra, Erik Prabowo, Hashim Talib Hashim, Maytham Al-Juaifari Al-Sader, Sohei Satoi, Khaled Obeidat, Maram Mohsen, Ho-Seong Han, Mohamad Khalife, Muhammed Elhadi, Audrius Dulskas, Jin Bong, Shahi Ghani, Alejandro Eduardo Padilla, Javier Melchor-Ruan, Sarnai Erdene, Amine Benkabbou, Pueya Nashidengo, Jonathan Koea, Ademola Adeyeye, Olusegun Alatise, Sami Ullah, Mustafa Abu Jayyab, Sarah Amro, Walaa Mohammed Alnammourah, Catherine The, Michał Pędziwiatr, Wojciech Polkowski, Sorin Barbu, Aleksandar Karamarkovic, Daniel Galun, Brian Goh, Blaž Trotovšek, Jones Omoshoro-Jones, Benedetto Ielpo, Abdelfatah Abdelmageed, Per Sandström, Alessandra Cristaudi, Beat Gloor, Christoph Kuemmerli, Alaa Hamdan Tishreen, Mohammad Karam Chaaban, Chien Hui Wu, Po-Chih Yang Fu Jen, Ammar Houssem, Oussama Baraket, Ahmet Çoker, Mark Taylor, Nigel Jamieson, Satheesh Iype, Emmanouil Giorgakis, Motaz Qadan, Sabha Ganai, Hamza Al-Naggar, Onesai Chihaka, all Pancreasgroup.org collaborators, Amor El Behi, Anisse Tidjane, Ilhem Ouahab, Souad Bouaoud, Mounira Rais, Meriem Abdoun, Amel Ouyahia, Lucas Mc Cormack, Martin de Santibanes, Pablo Barros Schelotto, Shantanu Joglekar, Sivakumar Gananadha, Kat Hall, Russell Hodgson, Alistair Rowcroft, Lynn Chong, Kalpesh Shah, Stanley Chen, David Burnett, Zi Qin Ng, Christos Apostolou, Peter Kornprat, Radoslava Stoyanova, Aliaksei Shcherba, Bert Van den Bossche, Vera Hartman, Filip Gryspeerdt, Frederik Berrevoet, Sébastien Strypstein, Rinaldo Pinto, Gustavo Coelho, Matheus Militz, Pablo Rodrigues, Orlando Torres, Narimã Marques, Adriano Sampaio, Panche Krastev, Mihail Slavchev, Ivelin Takorov, Nikola Vladov, Vassil Mihaylov, Evgeni Nikolaev, Daniel Kostov, Pablo Serrano, Cristian Diaz, Alejandro Brañes, Lei Cai, Yiming Chen, Ivan Štironja, Tomislav Bubalo, Martin Loveček, Pavel Zaruba, Andrej Nikov, Almoatazbellah Attalla, Mosaab Tayiawi, Muneera Alboridy, Mohamed Mourad, Mostafa Elkeleny, Yousef Tanas, Hajer Ealreibi, Mohamed Abd El Moneam, Mohammed Hamouda, Ahmed Nafea, Mohamed Abdelalemm, Ahmed Mansour, Marina Farag, Mohamed Salah, Mohamed Abdelkarem, Hatem Sayed, Sherein Diab, Mira Gobran, Abdelrahman Fahim, Ahmed K. Awad, Sherif Abdelmawgoud, Emad Alazab, Mostafa Nagy, Islam Metwally, Ahmed Shehta, Ahmed Monier, Selmy Awad, Mohammed Omar, Feras Alsabbagh, Joonas Kauppila, Minna Nortunen, Heikki Huhta, Raffaele Brustia, Stéphanie Truant, Guillaume Piessen, Aurélien Dupré, Francois-Regis Souche, Natalia Savala, Renato Lupinacci, Sébastien Gaujoux, Nicolas Goasguen, Morgan Anyla, Lilian Schwarz, Fabio Giannone, Zaza Demetrashvili, Isabel Bartella, Ioannis Pozios, Orlin Belyaev, Dirk Bulian, Christian Praetorius, Sandra Korn, Maximilian Brunner, Elena Mazzella, Martin Reichert, Jochen Gaedcke, Ulrich Ronellenfitsch, Tim Reese, Kim Wagner, Pasquale Scognamiglio, Faik Uzunoglu, Jakob Izbicki, Benjamin Struecker, Dimitrios Kardassis, Silvio Nadalin, Georgios Makridis, Nikolaos Tsoukalas, Aristotelis Kechagias, Argyrios Ioannidis, Nikolaos Arkadopoulos, Dimitrios Papakonstantinou, Nikolaos Michalopoulos, Panagiotis Kokoropoulos, Pantelis Vassiliu, Dimitrios Stergiou, Maria Sotiropoulou, Nefeli Tomara, Panagiotis Dorovinis, George Tzimas, Dimitrios Schizas, Alexandros Papalampros, Andreas Polydorou, Georgios Fragulidis, Konstantinos Toutouzas, Antonios Vezakis, Konstantinos Bramis, Dimitrios Korkolis, Evangelos Fradelos, Georgios Glantzounis, Dimitrios Magouliotis, Grigorios Christodoulidis, Francesk Mulita, Elissaios Kontis, Vasileios Papaziogas, Dimitris Giakoustidis, Anastasios Katsourakis, Achilleas Νtinas, Panagiotis Petras, Attila Bursics, Andras Vereczkei, Sreenivasan Karuparthi, Govind Purushothaman, Rahul Gupta, Kaushal Yadav, Sundeep Jain, Jeewan Ram Vishnoi, Sanjeev Misra, Vaibhav Varshney, Kshaunish Das, Somnath Chattopadhyay, Jayapala Reddy Velagala, Varun Bansal, Induchoodan S, Tarun Kumar, Erik Prabowo, Hashim Talib Hashim, Maytham Al-Juaifari, Eran Sadot, Marco Vivarelli, Valeria Andriola, Michele Ciola, Mario Virgilio Papa, Adelmo Antonucci, Diego Sasia, Valentina Testa, Lapo Bencini, Tommaso Nelli, Fabrizio D’Acapito, Carlo Alberto Pacilio, Giacomo Carganico, Raffaele De Rosa, Stefano D’Ugo, Edoardo Saladino, Alfonso Recordare, Michele Mazzola, Vincenzo Mazzaferro, Alessandro Zerbi, Domenico Tamburrino, Caterina Baldi, Marco Stella, Fabrizio Di Benedetto, Fabio Uggeri, Alessandro Iacomino, Gianluca Rompianesi, Andrea Belli, Lucia Moletta, Francesca Tolin, Mario Giuffrida, Lorenzo Cobianchi, Paolo Regi, Luca Morelli, Emanuele Federico Kauffmann, Enrico Pinotti, Felice Giuliante, Alessandro Coppola, Tommaso Maria Manzia, Alberto Porcu, Claudio Feo, Teresa Perra, Francesco Ciarleglio, Simone Novello, Alessandro Ferrero, Sergio Intini, Giorgio Querini, Andrea Ruzzenente, Tommaso Campagnaro, Simone Conci, Sara Napetti, Alice Frontali, Daisuke Hashimoto, Ippei Matsumoto, Hiromitsu Maehira, Minoru Tanabe, Subhi Alissawi, Aiman Obed, Hebah Rababa, Khayry Al-Shami, Mohamed Alsabah, Saeed Shumrakh, Abdulaziz Al-Samawi, Almu’Atasim Khamees, Ildar Fakhradiyev, Ho-Seong Han, Kristaps Atstupens, Walid Faraj, Mohamad Khalife, Fatoom Alowjali, Abdulhadi Alshatshat, Aihab Benamwor, Eman Younes, Dania Burgan, Marwa Morgom, Muhannud Binnawara, Entisar Alshareea, Sultan Ahmeed Ahmeed, Eman Othman, Ahmed Gerwash, Osama Salem, Wegdan Khalil, Eman Abdulwahed, Mohamed Alharari, Tomas Vanagas, Vitalijus Eismontas, Jonas Jurgaitis, Algirdas Slepavicius, Vytenis Mikutaitis, Mindaugas Kvietkauskas, Edoardo Rosso, Andee Dzulkarnaen Zakaria, Ian Chik, Jin Bong, Carlos Florez Zorrilla, Sarnai Erdene, Moniba Korch, Iltimass Gouazar, Badr Serji, Aziz Zentar, Amine Benkabbou, Reda Elhassouni, Sabrillah Echiguer, Mohammed Anass Majbar, Abdulrashid Pueya Nashidengo, Paleswan Joshi Lakhey, John Windsor, Michael Jen Jie Chu, Peter Johnston, Vanshay Bindra, Andrea Cross, Ashok Gunawardene, Fraser Welsh, Olusegun Alatise, Jibran Abbasy, Tayyab Siddiqui, Mohammad Asghar, Mustafa Abu Jayyab, Qusai Zreqat, Rawand Titi, Fatima Manasrah, Ahlam Hammoudeh, Guillermo Coayla, Catherine Teh, Cenon Alfonso, Marta Flisińska, Justyna Rymarowicz, Marek Sierzega, Wojciech Ciesielski, Oliwia Grząsiak, Patrycja Szewczyk, Krzysztof Szwedziak, Wojciech Korcz, Oskar Kornasiewicz, Emanuel Vigia, Raluca Bievel Radulescu, Traian Dumitrascu, Mara Mardare, Octav Ginghina, Iulian Brezean, Sorin Petrea, Adrian Bartos, Raluca Bodea, Sergiu Matei, Ana-Maria Musina, Natalia Velenciuc, Alexander Belyaev, Denis Mizgirev, Andrey Litvin, Ivan Semenenko, Arkady Bedzhanyan, Nikolay Bagmet, Ayrat Kaldarov, Denis Kuchin, Evgeniy Drozdov, Mahir Gachabayov, Mohammed Alharthi, Aleksandar Bogdanovic, Daniel Galun, Stefan Kmezic, Dragana Arbutina, Mihailo Bezmarevic, Jovan Juloski, Mladjan Protic, Brian Goh, Blaž Trotovšek, Emil Loots, Jones Omoshoro-Jones, Manuel Marcello, Jose Ramia, Maria Del Mar Rico-Morales, Fabio Ausania, Ana Belen Martin Arnau, Benedetto Ielpo, Esther Pilar Santos, Patricia Ruiz, Laia Falgueras, Farah Al Shwely, Juli Busquets, Iago Justo, Jana Dziakova, Marcello Di Martino, Miguel Ángel Suárez-Muñoz, Lorena Solar García, Juan Jose Segura-Sampedro, Fernando Rotellar, Luis Muñoz-Bellvis, Valle Vera, María García Domingo, Carlos Domingo-Del Pozo, Cristina Ballester Ibáñez, Dimitri Dorcaratto, Mario Rodriguez-Lopez, Alejandro Serrablo, Hytham Hamid, Abdelfatah Abdelmageed, Per Sandström, Linda Lundgren, Bobby Tingstedt, Caroline Williamsson, Bodil Andersson, Ernesto Sparrelid, Christoph Kuemmerli, Beat Moeckli, Alessandra Cristaudi, Fariba Abbassi, Jan Schmidt, Stefan Gutknecht, Jan Philipp Jonas, Mais Alhashemi, Zain Douba, Ahmad Kayali, Oula Azizeh, Omar Al-Abed, Aya Abdulmonem, Ahmad Alhouri, Hasan Al Houri, Alnour Suliman, Bashar Haj Hassan, Alaa Hamdan, Ming-Chin Yu, Hanen Bouaziz, Ali Kchaou, Ammar Houssem, Mesut Tez, Mustafa Kerem, Hüseyin Bayhan, Ulaş Aday, Alpen Gumusoglu, Husnu Aydin, Fatema Hanefa, Ali Emre Atici, Hanife Ulgur, Ismail Sert, Semra Demirli Atici, Elif Colak, Denys Skoryi, Kostiantyn Kopchak, Oleksandr Kvasivka, Valeriia Sumarokova, James Skipworth, William Lim, Ahmed Mohamed, Pascale Tinguely, Hemant Kocher, Amjad Khalil, Nicola de’ Liguori Carino, Fahed Gareb, Pandanaboyana Sanjay, Krishnakumure Patel, Carlo Ceresa, Thomas Russell, Matt Mortimer, Megan Sulciner, Thomas Clancy, Martina Nebbia, Rachel Thompson, Dimitrios Moris, Jennifer Gnerlich, Richard Spencer-Cole, Derek Krinock, Tamara Osborn, Hailey Hardgrave, Joe Nigh, Beth Schrope, Ryan Lamm, Harish Lavu, Wilbur Bowne, Jonathan Sham, Paulo Martins, Aisha Albar, Fatima Al-Eryani, Hamza Al-Naggar, Rafat Al-Saban

**Affiliations:** 1 Department of HPB Surgery and Liver Transplant, Royal Free Hospital NHS Foundation Trust, London, UK; 2 Centre for Evidence-Based Medicine, Nuffield Department of Primary Care Health Sciences, University of Oxford, Radcliffe Observatory Quarter, Oxford, UK; 3 Department for Continuing Education, University of Oxford, Oxford, UK; 4 King Faisal Specialist Hospital & Research Centre, Organ Transplant Centre of Excellence, Riyadh, Saudi Arabia; 5 Department of Surgical Biotechnology, University College London, London, UK

**Correction to: Ann Surg Oncol** 10.1245/s10434-025-17911-8

In the original online version of this article, Cristina Ferrone was inadvertently omitted as the Pancreasgroup.org Chief Investigator.

The original article was corrected.

