# Supplementary material for: Analysis of Short-Term Outcomes in Pancreatic Surgery with Vascular Resection from a Prospective Multicenter Global Study
Source: Ann Surg Oncol. 2025 Aug 26;32(12):8870–80. doi: 10.1245/s10434-025-17911-8 (PMC12534231; doi:10.1245/s10434-025-17911-8)
Supplement: Supplementary file 1 — Supplementary file1 (DOCX 75 KB) [file 10434_2025_17911_MOESM1_ESM.docx]

**Appendix – PancreasGroup.org Group Authorship**

**PancreasGroup.org Committees**

**Chief Investigators:** Giuseppe Kito Fusai, Royal Free Hospital & University College London, UK. Cristina Ferrone, Cedars Sinai, Los Angeles, USA.

**Co-Chief Investigator:** Dimitri Aristotle Raptis, King Faisal Specialist Hospital and Research Center, Riyadh, Saudi Arabia. Pascale Tinguely, Royal Free Hospital, London, UK. Camila Hidalgo Salinas, University of Oxford, Oxford, UK.

**Scientific Committee:**

Mohamed Abu Hilal, University Hospital Southampton, UK. Claudio Bassi, University Hospital of Verona, Italy. Marc Besselink, University of Amsterdam, Netherlands. Kevin Conlon, Trinity College Dublin, Ireland. Brian Davidson, University College London, UK. Marco Del Chiaro, University of Colorado Anschutz Medical Campus, USA. Christos Dervenis, Metropolitan Hospital Athens, Greece. Massimo Falconi, San Raffaele Hospital, Milano, Italy. Thilo Hackert, University of Heidelberg, Germany. Ewen Harrison, University of Edinburgh, UK. Shailesh V. Shrikhande, Tata Memorial Hospital, Mumbai, India. Ajith Siriwardena, Manchester University, UK. Martin Smith, University of the Witwatersrand, Johannesburg, South Africa. Christopher Wolfgang, New York University Langone Health, New York, USA

**Management Committee**:

Aditya Borakati, Royal Free Hospital, London, UK. Deniz Balci, Ankara University Hospital, Turkey. Camila Hidalgo Salinas, Royal Free Hospital, London, UK. Nikolaos Machairas, Royal Free Hospital, London, UK. Giovanni Marchegiani, University Hospital of Verona, Italy. Atsushi Oba, University of Colorado Anschutz Medical Campus, USA. Christian Oberkofler, University Hospital Zurich, Switzerland. Ioannis Passas, Metropolitan Hospital Athens, Greece. Reena Ravikumar, Royal Infirmary of Edinburgh, UK. Patricia Sánchez Velázquez, University Hospital del Mar, Barcelona, Spain. Martin de Santibañes, Italian Hospital of Buenos Aires, Argentina. Andreas Anton Schnitzbauer, University Hospital Frankfurt, Germany. Fiammetta Soggiu, Royal Free Hospital, London, UK. Domenico Tamburrino, San Raffaele Hospital, Milano, Italy. Pascale Tinguely, Royal Free Hospital, London, UK. Alice Wei, Memorial Sloan Kettering Cancer, New York, USA. Marinos Zachiotis, University of Patras, Greece.

**Country Leaders:**

**Algeria:** Kamel Bentabak, Pierre and Marie Curie Cancer Center, Algiers; Salah Eddine Kacimi, Faculty of Medicine, University of Tlemcen Tlemcen. **Australia:** Mehrdad Nikfarjam, University of Melbourne, Melbourne. **Belarus**: Aliaksei Shcherba, Minsk medical Center for Surgery, Transplantation and Hematology Minsk. **Belgium:** Gregory Sergeant, Jessa Ziekenhuis Hasselt. **Brazil:** Gustavo Coelho, Federal University of Ceara Fortaleza. Orlando Torres, Maranhão Federal University Sao Luis. **Bulgaria**: Nikolay Belev, UMHAT-"Eurohospital", Medical Training and Simulation Centre, MU-Plovdiv; Burundi Fabrice, Muhezagiro University of Burundi, Bujumbura. **Canada:** Ephraim Tang, London Health Sciences Centre - Western University London, Ontario; Janet Martin, Western University London. **Chile**: Christian Diaz, Hospital Dr Sotero del Rio, Santiago; Nicolas Devaud Instituto Oncologico Fundacion Arturo Lopez Perez (FALP) Santiago. **China**: Kongyuan Wei, Heidelberg University Hospital/ Chinese PLA General Hospital, Beijing; Maher Hendi, Zhejiang University School of Medicine, Sir Ren Ren Shaw Hospital, Hangzhou. **Croatia**: Danko Mikulic University Hospital Merkur Zagreb. **Cyprus**: Nikolaos Gouvas, University of Cyprus, Nicosia. **Czech Republic**: Andrej Nikov, University Hospital Kralovske Vinohrady, Prague. **Egypt**: Dalia Fathallah, Alexandria University Main Hospital, Alexandria; Mahmoud Saad, Assiut Faculty of Medicine, Assiut. **Estonia**: Olav Tammik, University Clinic of Tartu, Tartu. **Finland:** Heikki Huhta, Oulu University Hospital, Oulu. **France:** Laurent Sulpice, University Hospital Rennes, Rennes; Renato Lupinacci, Ambroise Pare Hospital, Paris-Saclay University, Paris. **Germany**: Gregor Stavrou, Klinikum Saarbrücken. **Greece** Evangelos Felekouras, National and Kapodistrian University of Athens; Vasileios Papaziogas, Aristotle University of Thessaloniki. **India**: Sanjeev Misra, All India Institute of Medical Sciences Jodhpur. **Indonesia**: Erik Prabowo Diponegoro University / Kariadi General Hospital, Semarang. **Iraq**: Hashim Talib Hashim University of Baghdad, College of Medicine, Baghdad; Maytham Al-Juaifari Al-Sader, Teaching Hospital, An Najaf. **Japan**: Sohei Satoi, Kansai Medical University Hirakata. **Jordan**: Khaled Obeidat, Jordan University of Science and Technology, Irbid; Maram Mohsen, Jordan University Hospital, Amman. Republic of **Korea,** Ho-Seong Han, Seoul National University Bundang Hospital, Seongnam. **Lebanon**: Mohamad Khalife, American University of Beirut. **Libya**: Muhammed Elhadi, Faculty of Medicine, University of Tripoli. **Lithuania**: Audrius Dulskas, National Cancer Institute Vilnius. **Malaysia**: Jin Bong Sunway Medical Center Petaling Jaya. **Maldives:** Shahi Ghani, Tree Top Hospital, Male'. Mexico: Alejandro Eduardo Padilla, Rosciano National Cáncer Institute, Mexico City; Javier Melchor-Ruan, National Cancer Institute Mexico. **Mongolia**: Sarnai Erdene, Mongolian National University of Medical Sciences, Ulaanbaatar. **Morocco**: Amine Benkabbou, National Institute of Oncology, Mohammed V University in Rabat. **Namibia**: Pueya Nashidengo, Windhoek Central Academic Hospital/University of Namibia School of Medicine, Windhoek. **New Zealand**: Jonathan Koea, North Shore Hospital, Auckland. **Nigeria** Ademola Adeyeye, Afe Babalola University Ado-Ekiti(ABUAD) Ado-Ekiti; Olusegun Alatise, Obafemi Awolowo University, Ile Ife. **Pakistan:** Sami Ullah Services Hospital Lahore Lahore. Palestine, Mustafa Abu Jayyab Alshifaa Hospital, Gaza; Sarah Amro, Alia Govermental Hospital, Hebron; Walaa Mohammed Alnammourah, Alia Governmental Hospital, Hebron. **Philippines**: Catherine The, Makati Medical Center, Makati. **Poland**: Michał Pędziwiatr, Jagiellonian University Medical College, Kraków; Wojciech Polkowski, Medical University of Lublin. **Romania**: Sorin Barbu, "Iuliu Hatieganu" University of Medicine & Pharmacy, Cluj-Napoca **Serbia**: Aleksandar Karamarkovic, Surgical Clinic "Nikola Spasic", University Clinical Center "Zvezdara", Belgrade, Faculty of Medicine University of Belgrade; Daniel Galun, Clinic for Digestive Surgery, Clinical Center of Serbia, Belgrade. **Singapore**: Brian Goh, Singapore General Hospital, Singapore. **Slovenia**: Blaž Trotovšek, University Medical Centre Ljubljana. **South** **Africa**: Jones Omoshoro-Jones, Chris Hani Baragwanath Academic Hospital/University of the Witwatersrand, Johannesburg. **Spain**: Benedetto Ielpo, Hospital del Mar, Barcelona. **Sudan**: Abdelfatah Abdelmageed, Omdurman Islamic University, Khartoum. **Sweden**: Per Sandström, University Hospital of Linkoping. **Switzerland**: Alessandra Cristaudi, Ospedale Regionale di Lugano; Beat Gloor, Inselspital, University Bern; Christoph Kuemmerli, Clarunis, University Centre for Gastrointestinal and Liver Diseases, St. Clara Hospital and University Hospital, Basel. **Syria**: Alaa Hamdan Tishreen, University Hospital Lattakia; Mohammad Karam Chaaban Al-Mouwasat University Hospital, Damascus. **Taiwan**: Chien Hui Wu, National Taiwan University Hospital, Taipei; Po-Chih Yang Fu Jen, Catholic University Hospital, New Taipei City. **Tunisia**: Ammar Houssem Sahloul Hospital, Sousse; Oussama Baraket, Hopital Habib Bouguetfa de Bizerte, University Tunis El Labar Bizerte. **Turkey**: Ahmet Çoker Ege University, Izmir HPB Clinic, Izmir. **United Kingdom**: Mark Taylor, Belfast Health and Social Care Trust, Belfast; Nigel Jamieson, University of Glasgow; Satheesh Iype, Royal Free Hospital London. **United States of America**: Emmanouil Giorgakis, UAMS Medical Center/ Winthrop P Rockefeller Cancer Institute, Little Rock; Motaz Qadan, Massachusetts General Hospital, Boston; Sabha Ganai, University of North Dakota, Sanford Health, Fargo. **Yemen**: Hamza Al-Naggar, Faculty of Medicine - Sana'a University, Sana'a. **Zimbabwe**: Onesai Chihaka, University of Zimbabwe, Harare.

**Collaborators:**

| FIRST NAME | LAST NAME | INSTITUTION | CITY | COUNTRY |
| --- | --- | --- | --- | --- |
| Amor | El Behi | University Of Sousse | Alger | Algeria |
| Anisse | Tidjane | Ehu-1St November 1954, Departmenet Of Medicine, University Oran 1 | Oran | Algeria |
| Ilhem | Ouahab | Ferhat Abbas University, Sétif 1, Faculty Of Medicine. Chu Saadna Abdenour. | Setif | Algeria |
| Souad | Bouaoud | Ferhat Abbas University, Sétif 1, Faculty Of Medicine. Chu Saadna Abdenour. | Sétif | Algeria |
| Mounira | Rais | Ferhat Abbas University, Sétif 1, Faculty Of Medicine. Chu Saadna Abdenour. | Sétif | Algeria |
| Meriem | Abdoun | Ferhat Abbas University, Sétif 1, Faculty Of Medicine. Chu Saadna Abdenour. | Sétif | Algeria |
| Amel | Ouyahia | Ferhat Abbas University, Sétif 1, Faculty Of Medicine. Chu Saadna Abdenour. | Sétif | Algeria |
| Lucas | Mc Cormack | Hospital Aleman Of Buenos Aires | Buenos Aires | Argentina |
| Martin | de Santibanes | Hospital Italiano De Buenos Aires | Buenos Aires | Argentina |
| Pablo | Barros Schelotto | Universitary Hospital Favaloro Foundation | Buenos Aires | Argentina |
| Shantanu | Joglekar | Box Hill Hospital | Burwood | Australia |
| Sivakumar | Gananadha | Canberra Hospital | Canberra | Australia |
| Kat | Hall | Austin Health / Knox Private Hospital | Melbourne | Australia |
| Russell | Hodgson | Northern Health | Melbourne | Australia |
| Alistair | Rowcroft | St Vincent'S Hospital Melbourne | Melbourne | Australia |
| Lynn | Chong | St Vincent'S Hospital Melbourne | Melbourne | Australia |
| Kalpesh | Shah | John Hunter Hospital | Newcastle | Australia |
| Stanley | Chen | John Hunter Hospital | Newcastle | Australia |
| David | Burnett | John Hunter Hospital | Newcastle | Australia |
| Zi Qin | Ng | Royal Perth Hospital | Perth | Australia |
| Christos | Apostolou | Sydney Adventist Hospital | Sydney | Australia |
| Peter | Kornprat | Medical University Graz | Graz | Austria |
| Radoslava | Stoyanova | Hospital Sisters Of Mercy | Vienna | Austria |
| Aliaksei | Shcherba | Minsk Medical Center For Surgery, Transplantation And Hematology | Minsk, Belarus | Belarus |
| Bert | Van den Bossche | A.S.Z. Aalst - Geraardsbergen - Wetteren | Aalst | Belgium |
| Vera | Hartman | Antwerp University Hospital | Edegem | Belgium |
| Filip | Gryspeerdt | University Hospital Ghent | Gent | Belgium |
| Frederik | Berrevoet | University Hospital Ghent | Gent | Belgium |
| Sébastien | Strypstein | Az Delta - Az St. Jan Brugge | Roeselare | Belgium |
| Rinaldo | Pinto | Hospital Santa Catarina De Blumenau | Blumenau | Brazil |
| Gustavo | Coelho | Federal University Of Ceara | Fortaleza | Brazil |
| Matheus | Militz | Hospital De Clínicas De Porto Alegre | Porto Alegre | Brazil |
| Pablo | Rodrigues | Ufcspa / Santa Casa Porto Alegre | Porto Alegre | Brazil |
| Orlando | Torres | Maranhão Federal University | Sao Luis | Brazil |
| Narimã | Marques | AC Camargo Cancer Center | São Paulo | Brazil |
| Adriano | Sampaio | Hospital Do Servidor Público Estadual De São Paulo | São Paulo | Brazil |
| Panche | Krastev | UMHAT Eurohospital | Plovdiv | Bulgaria |
| Mihail | Slavchev | UMHAT Eurohospital | Plovdiv | Bulgaria |
| Ivelin | Takorov | Military Medical Academy - Sofia | Sofia | Bulgaria |
| Nikola | Vladov | Military Medical Academy - Sofia | Sofia | Bulgaria |
| Vassil | Mihaylov | Military Medical Academy - Sofia | Sofia | Bulgaria |
| Evgeni | Nikolaev | Military Medical Academy - Varna | Varna | Bulgaria |
| Daniel | Kostov | Military Medical Academy - Varna | Varna | Bulgaria |
| Pablo | Serrano | McMaster University | Hamilton | Canada |
| Cristian | Diaz | Hospital Sotero del Río | Santiago | Chile |
| Alejandro | Brañes | Hospital Sotero del Río | Santiago | Chile |
| Lei | Cai | Chongqing General Hospital, University Of Chinese Academy Of Sciences | Chongqing | China |
| Yiming | Chen | The First Affiliated Hospital Of Dali University | Dali | China |
| Ivan | Štironja | University Hospital Merkur | Zagreb | Croatia |
| Tomislav | Bubalo | University Hospital Merkur | Zagreb | Croatia |
| Martin | Loveček | University Hospital Olomouc, Palacky University Olomouc; | Olomouc | Czech Republic (the) |
| Pavel | Zaruba | Military University Hospital Prague | Prague | Czech Republic (the) |
| Andrej | Nikov | University Hospital Kralovske Vinohrady Prague | Prague | Czech Republic (the) |
| Almoatazbellah | Attalla | Alexanderia University Hospital | Alexanderia | Egypt |
| Mosaab | Tayiawi | Alexanderia University Hospital | Alexandria | Egypt |
| Muneera | Alboridy | Alexanderia University Hospital | Alexandria | Egypt |
| Mohamed | Mourad | Alexanderia University Hospital | Alexandria | Egypt |
| Mostafa | Elkeleny | Alexanderia University Hospital | Alexandria | Egypt |
| Yousef | Tanas | Alexanderia University Hospital | Alexandria | Egypt |
| Hajer | Ealreibi | Alexanderia University Hospital | Alexandria | Egypt |
| Mohamed | Abd El Moneam | Alexanderia University Hospital | Alexandria | Egypt |
| Mohammed | Hamouda | Alexanderia University Hospital | Alexandria | Egypt |
| Ahmed | Nafea | Alexanderia University Hospital | Alexandria | Egypt |
| Mohamed | Abdelalemm | Alexanderia University Hospital | Alexandria | Egypt |
| Ahmed | Mansour | Alexanderia University Hospital | Alexandria | Egypt |
| Marina | Farag | Alexanderia University Hospital | Alexandria | Egypt |
| Mohamed | Salah | Al-Rajhi Liver Hospital | Assuit | Egypt |
| Mohamed | Abdelkarem | Assuit Universty Hospital | Assuit | Egypt |
| Hatem | Sayed | Ain Shams University Hospitals | Cairo | Egypt |
| Sherein | Diab | Ain Shams University Hospitals | Cairo | Egypt |
| Mira | Gobran | Ain Shams University Hospitals | Cairo | Egypt |
| Abdelrahman | Fahim | Ain Shams University Hospitals | Cairo | Egypt |
| Ahmed K. | Awad | Ain Shams University Hospitals | Cairo | Egypt |
| Sherif | Abdelmawgoud | Ain Shams University Hospitals | Cairo | Egypt |
| Emad | Alazab | Ain Shams University Hospitals | Cairo | Egypt |
| Mostafa | Nagy | Ain Shams University Hospitals | Cairo | Egypt |
| Islam | Metwally | Mansoura University | Mansoura | Egypt |
| Ahmed | Shehta | Mansoura University | Mansoura | Egypt |
| Ahmed | Monier | Mansoura University | Mansoura | Egypt |
| Selmy | Awad | Mansoura University | Mansoura | Egypt |
| Mohammed | Omar | Faculty Of Medicine, South Valley University | Qena | Egypt |
| Feras | Alsabbagh | Zagazig University | Zagazig | Egypt |
| Joonas | Kauppila | Oulu University Hospital | Oulu | Finland |
| Minna | Nortunen | Oulu University Hospital | Oulu | Finland |
| Heikki | Huhta | Oulu University Hospital | Oulu | Finland |
| Raffaele | Brustia | Henri Mondor University Hospital | Créteil | France |
| Stéphanie | Truant | Hôpital Huriez, Chu Lille | Lille | France |
| Guillaume | Piessen | Hôpital Huriez, Chu Lille | Lille | France |
| Aurélien | Dupré | Centre Leon Berard | Lyon | France |
| Francois-Regis | Souche | Chu Montpellier | Montpellier | France |
| Natalia | Savala | Hôpital De L' Archet, Nice | Nice | France |
| Renato | Lupinacci | Ambroise Pare Hospital, Paris-Saclay University | Paris | France |
| Sébastien | Gaujoux | APHP | Paris | France |
| Nicolas | Goasguen | Hopital Diaconesses/Croix Saint Simon | Paris | France |
| Morgan | Anyla | Hôpital Pitié-Slapétrière | Paris | France |
| Lilian | Schwarz | Rouen University Hospital | Rouen | France |
| Fabio | Giannone | Nouvel Hôpital Civil | Strasbourg | France |
| Zaza | Demetrashvili | Tbilisi State Medical University | Tbilisi | Georgia |
| Isabel | Bartella | Marienhospital Aachen | Aachen | Germany |
| Ioannis | Pozios | Charité - Universitätsmedizin Berlin | Berlin | Germany |
| Orlin | Belyaev | St. Josef Hospital, Ruhr University Bochum | Bochum | Germany |
| Dirk | Bulian | Cologne-Merheim Medical Center, Witten/Herdecke University | Cologne | Germany |
| Christian | Praetorius | University Hospital Carl Gustav Carus | Dresden | Germany |
| Sandra | Korn | University Hospital Carl Gustav Carus | Dresden | Germany |
| Maximilian | Brunner | University Hospital Erlangen | Erlangen | Germany |
| Elena | Mazzella | Uniklinik Frankfurt | Frankfurt Am Main | Germany |
| Martin | Reichert | University Hospital Of Giessen | Giessen | Germany |
| Jochen | Gaedcke | University Medical Center Göttingen | Göttingen | Germany |
| Ulrich | Ronellenfitsch | University Hospital Halle (Saale) | Halle (Saale) | Germany |
| Tim | Reese | Asklepios Hospital Barmbek | Hamburg | Germany |
| Kim | Wagner | Asklepios Hospital Barmbek | Hamburg | Germany |
| Pasquale | Scognamiglio | University Medical Center Hamburg-Eppendorf | Hamburg | Germany |
| Faik | Uzunoglu | University Medical Center Hamburg-Eppendorf | Hamburg | Germany |
| Jakob | Izbicki | University Medical Center Hamburg-Eppendorf | Hamburg | Germany |
| Benjamin | Struecker | Universitätsklinikum Münster (Ukm) | Münster | Germany |
| Dimitrios | Kardassis | Klinikum Saarbrücken | Saarbruecken | Germany |
| Silvio | Nadalin | University Hospital Tübingen | Tübingen | Germany |
| Georgios | Makridis | St. Josefs-Hospital Wiesbaden | Wiesbaden | Germany |
| Nikolaos | Tsoukalas | 401 General Military Hospital Of Athens | Athens | Greece |
| Aristotelis | Kechagias | Athens Bioclinic Hospital | Athens | Greece |
| Argyrios | Ioannidis | Athens Medical Center | Athens | Greece |
| Nikolaos | Arkadopoulos | University General Hospital Attikon | Athens | Greece |
| Dimitrios | Papakonstantinou | University General Hospital Attikon | Athens | Greece |
| Nikolaos | Michalopoulos | University General Hospital Attikon | Athens | Greece |
| Panagiotis | Kokoropoulos | University General Hospital Attikon | Athens | Greece |
| Pantelis | Vassiliu | University General Hospital Attikon | Athens | Greece |
| Dimitrios | Stergiou | Evangelismos General Hospital | Athens | Greece |
| Maria | Sotiropoulou | Evangelismos General Hospital | Athens | Greece |
| Nefeli | Tomara | Laiko General Hospital of Athens | Athens | Greece |
| Panagiotis | Dorovinis | Laiko General Hospital of Athens | Athens | Greece |
| George | Tzimas | Hygeia Hospital | Athens | Greece |
| Dimitrios | Schizas | National And Kapodistrian University Of Athens | Athens | Greece |
| Alexandros | Papalampros | National And Kapodistrian University Of Athens | Athens | Greece |
| Andreas | Polydorou | National And Kapodistrian University Of Athens | Athens | Greece |
| Georgios | Fragulidis | National And Kapodistrian University Of Athens | Athens | Greece |
| Konstantinos | Toutouzas | National And Kapodistrian University Of Athens | Athens | Greece |
| Antonios | Vezakis | National And Kapodistrian University Of Athens | Athens | Greece |
| Konstantinos | Bramis | National And Kapodistrian University Of Athens | Athens | Greece |
| Dimitrios | Korkolis | Saint Savvas Cancer Hospital | Athens | Greece |
| Evangelos | Fradelos | Saint Savvas Cancer Hospital | Athens | Greece |
| Georgios | Glantzounis | Ioannina University Hospital And School Of Medicine, University Of Ioannina | Ioannina | Greece |
| Dimitrios | Magouliotis | University Hospital Of Larissa | Larissa | Greece |
| Grigorios | Christodoulidis | University Hospital Of Larissa | Larissa | Greece |
| Francesk | Mulita | University Hospital Of Patras | Patras | Greece |
| Elissaios | Kontis | Metaxa Cancer Hospital | Pireas | Greece |
| Vasileios | Papaziogas | Aristotle University Of Thessaloniki | Thessaloniki | Greece |
| Dimitris | Giakoustidis | Aristotle University Of Thessaloniki | Thessaloniki | Greece |
| Anastasios | Katsourakis | General Hospital of Thessaloniki "O Agios Dimitrios" | Thessaloniki | Greece |
| Achilleas | Νtinas | Interbalkan Medical Center | Thessaloniki | Greece |
| Panagiotis | Petras | Ippokratio General Hospital Of Thessaloniki | Thessaloniki | Greece |
| Attila | Bursics | Uzsoki Hospital | Budapest | Hungary |
| Andras | Vereczkei | Clinical Center, Medical School University Of Pécs | Pécs | Hungary |
| Sreenivasan | Karuparthi | Sri Ramachandra Institute Of Higher Education And Research | Chennai | India |
| Govind | Purushothaman | Stanley Medical College | Chennai | India |
| Rahul | Gupta | Synergy Institute Of Medical Sciences | Dehradun | India |
| Kaushal | Yadav | W Pratiksha Hospital, Gurgaon | Gurugram | India |
| Sundeep | Jain | Ck Birla Hospitals/ Rbh Jaipur, India | Jaipur | India |
| Jeewan Ram | Vishnoi | All India Institute Of Medical Sciences, Jodhpur | Jodhpur | India |
| Sanjeev | Misra | All India Institute Of Medical Sciences, Jodhpur | Jodhpur | India |
| Vaibhav | Varshney | All India Institute Of Medical Sciences, Jodhpur | Jodhpur | India |
| Kshaunish | Das | Institute Of Postgraduate Medical Education & Research | Kolkata | India |
| Somnath | Chattopadhyay | Kokilaben Dhirubhai Ambani Hospital | Mumbai | India |
| Jayapala Reddy | Velagala | Kokilaben Dhirubhai Ambani Hospital | Mumbai | India |
| Varun | Bansal | Seth G.S Medical College And K.E.M Hospital | Mumbai | India |
| Induchoodan | S | Government Medical College, Thiruvananthapuram | Thiruvananthapuram | India |
| Tarun | Kumar | Institute Of Medical Sciences, Banaras Hindu University, Varanasi | Varanasi | India |
| Erik | Prabowo | Diponegoro University / Kariadi General Hospital | Semarang | Indonesia |
| Hashim Talib | Hashim | University Of Baghdad, College Of Medicine | Baghdad | Iraq |
| Maytham | Al-Juaifari | Al-Sader Teaching Hospital, Najaf | Najaf | Iraq |
| Eran | Sadot | ​Rabin Medical Center | Petah Tikva | Israel |
| Marco | Vivarelli | Marche Polytechnic University | Ancona | Italy |
| Valeria | Andriola | Policlinico Di Bari | Bari | Italy |
| Michele | Ciola | Regional Hospital Bolzano/Bozen | Bolzano/Bozen | Italy |
| Mario Virgilio | Papa | Aorn Caserta | Caserta | Italy |
| Adelmo | Antonucci | Sant'Anna Hospital | Como | Italy |
| Diego | Sasia | Santa Croce And Carle Hospital, Cuneo | Cuneo | Italy |
| Valentina | Testa | Santa Croce And Carle Hospital, Cuneo | Cuneo | Italy |
| Lapo | Bencini | Azienda Ospedaliero-Universitaria Careggi | Florence | Italy |
| Tommaso | Nelli | Ospedale San Giovanni Di Dio | Florence | Italy |
| Fabrizio | D'Acapito | Ausl Romagna, Morgagni-Pierantoni Hospital | Forlì | Italy |
| Carlo Alberto | Pacilio | Ausl Romagna, Morgagni-Pierantoni Hospital | Forlì | Italy |
| Giacomo | Carganico | Ospedale San Martino | Genova | Italy |
| Raffaele | De Rosa | University Of Genoa | Genova | Italy |
| Stefano | D'Ugo | Division Of Hpb Surgery - "Vito Fazzi" Hospital | Lecce | Italy |
| Edoardo | Saladino | Azienda Ospedaliera Papardo | Messina | Italy |
| Alfonso | Recordare | Dell'Angelo Hospital | Mestre | Italy |
| Michele | Mazzola | Asst Grande Ospedale Metropolitano Niguarda | Milan | Italy |
| Vincenzo | Mazzaferro | Fondazione IRCCS Istituto Nazionale Tumori | Milan | Italy |
| Alessandro | Zerbi | Humanitas Research Hospital | Milan | Italy |
| Domenico | Tamburrino | IRCCS San Raffaele Hospital, Vita-Salute University | Milan | Italy |
| Caterina | Baldi | San Carlo Borromeo Hospital, University Of Milano | Milan | Italy |
| Marco | Stella | San Carlo Borromeo Hospital, University Of Milano | Milan | Italy |
| Fabrizio | Di Benedetto | University Of Modena And Reggio Emilia | Modena | Italy |
| Fabio | Uggeri | IRCCS Foundation San Gerardo dei Tintori | Monza | Italy |
| Alessandro | Iacomino | Ospedale Antonio Cardarelli | Naples | Italy |
| Gianluca | Rompianesi | Federico Ii University Hospital | Naples | Italy |
| Andrea | Belli | Istituto Nazionale Tumori - Irccs - Fondazione G. Pascale | Naples | Italy |
| Lucia | Moletta | University Of Padova | Padova | Italy |
| Francesca | Tolin | Veneto Institute Of Oncology Iov – Irccs | Padova | Italy |
| Mario | Giuffrida | Parma University Hospital | Parma | Italy |
| Lorenzo | Cobianchi | University Of Pavia | Pavia | Italy |
| Paolo | Regi | Pederzoli Hospital | Peschiera Del Garda | Italy |
| Luca | Morelli | University Of Pisa | Pisa | Italy |
| Emanuele Federico | Kauffmann | University Of Pisa | Pisa | Italy |
| Enrico | Pinotti | Policlinico San Pietro | Ponte San Pietro | Italy |
| Felice | Giuliante | Fondazione Policlinico Universitario Agostino Gemelli IRCCS | Rome | Italy |
| Alessandro | Coppola | Fondazione Policlinico Universitario Campus Bio-Medico | Rome | Italy |
| Tommaso Maria | Manzia | Tor Vergata University of Rome | Rome | Italy |
| Alberto | Porcu | Azienda Ospedaliero Universitaria di Sassari | Sassari | Italy |
| Claudio | Feo | Azienda Ospedaliero Universitaria di Sassari | Sassari | Italy |
| Teresa | Perra | Azienda Ospedaliero Universitaria di Sassari | Sassari | Italy |
| Francesco | Ciarleglio | Santa Chiara Hospital - APSS | Trento | Italy |
| Simone | Novello | Iv Chirurgia, Ospedale Di Treviso; Università Di Padova | Treviso | Italy |
| Alessandro | Ferrero | Ospedale Mauriziano | Turin | Italy |
| Sergio | Intini | Università Degli Studi Di Udine - Asufc Ospedale Santa Maria Della Misericordia | Udine | Italy |
| Giorgio | Querini | Ospedale "Castelli", Verbania - Asl Vco | Verbania | Italy |
| Andrea | Ruzzenente | The Pancreas Institute, University of Verona | Verona | Italy |
| Tommaso | Campagnaro | The Pancreas Institute, University of Verona | Verona | Italy |
| Simone | Conci | The Pancreas Institute, University of Verona | Verona | Italy |
| Sara | Napetti | San Bortolo Hospital | Vicenza | Italy |
| Alice | Frontali | ASST Brianza - Vimercate Hospital | Vimercate, Milano | Italy |
| Daisuke | Hashimoto | Kansai Medical University | Hirakata, Osaka | Japan |
| Ippei | Matsumoto | Kindai University | Osaka-Sayama | Japan |
| Hiromitsu | Maehira | Shiga University Of Medical Science | Otsu | Japan |
| Minoru | Tanabe | Tokyo Medical And Dental University | Tokyo | Japan |
| Subhi | Alissawi | Al-Basheer Hospital | Amman | Jordan |
| Aiman | Obed | Jordan University Hospital | Amman | Jordan |
| Hebah | Rababa | Jordan University Hospital | Amman | Jordan |
| Khayry | Al-Shami | Jordan University Hospital | Amman | Jordan |
| Mohamed | Alsabah | King Abdullah University Hospital | Irbid | Jordan |
| Saeed | Shumrakh | King Abdullah University Hospital | Irbid | Jordan |
| Abdulaziz | Al-Samawi | King Abdullah University Hospital | Irbid | Jordan |
| Almu'Atasim | Khamees | Yarmouk University - Irbid | Irbid | Jordan |
| Ildar | Fakhradiyev | Kaznmu | Almaty | Kazakhstan |
| Ho-Seong | Han | Seoul National University Bundang Hospital | Seongnam | Korea (the Republic of) |
| Kristaps | Atstupens | Riga East Clinical University Hospital | Riga | Latvia |
| Walid | Faraj | American University Of Beirut | Beirut | Lebanon |
| Mohamad | Khalife | American University Of Beirut | Beirut | Lebanon |
| Fatoom | Alowjali | Banghazi Aljala Hospital | Banghazi | Libya |
| Abdulhadi | Alshatshat | National Cancer Institute | Misurata | Libya |
| Aihab | Benamwor | National Cancer Institute | Misurata | Libya |
| Eman | Younes | National Cancer Institute Of Sabratah | Sabratah | Libya |
| Dania | Burgan | National Cancer Institute, Sabratha - Libya | Sabratha | Libya |
| Marwa | Morgom | Alestiklal Hospital At Libya | Tripoli | Libya |
| Muhannud | Binnawara | Tripoli Central Hospital | Tripoli | Libya |
| Entisar | Alshareea | Tripoli Central Hospital | Tripoli | Libya |
| Sultan Ahmeed | Ahmeed | Tripoli Central Hospital | Tripoli | Libya |
| Eman | Othman | Tripoli Central Hospital | Tripoli | Libya |
| Ahmed | Gerwash | Tripoli Central Hospital | Tripoli | Libya |
| Osama | Salem | Tripoli Medical Center | Tripoli | Libya |
| Wegdan | Khalil | Tripoli Medical Center | Tripoli | Libya |
| Eman | Abdulwahed | Tripoli Medical Center | Tripoli | Libya |
| Mohamed | Alharari | Tripoli Medical Center | Tripoli | Libya |
| Tomas | Vanagas | Lithuanian University Of Health Sciences | Kaunas | Lithuania |
| Vitalijus | Eismontas | Klaipeda University Hospital | Klaipeda | Lithuania |
| Jonas | Jurgaitis | Klaipeda University Hospital | Klaipeda | Lithuania |
| Algirdas | Slepavicius | Klaipeda University Hospital | Klaipeda | Lithuania |
| Vytenis | Mikutaitis | Klaipeda University Hospital | Klaipėda | Lithuania |
| Mindaugas | Kvietkauskas | Vilnius University Hospital Santaros Klinikos | Vilnius | Lithuania |
| Edoardo | Rosso | Centre Hospitalier Du Luxembourg | Luxembourg | Luxembourg |
| Andee Dzulkarnaen | Zakaria | School Of Medical Sciences & Hospital, Universiti Sains Malaysia | Kota Bharu, Kelantan | Malaysia |
| Ian | Chik | Universiti Kebangsaan Malaysia Medical Centre | Kuala Lumpur | Malaysia |
| Jin | Bong | Sunway Medical Center | Subang Jaya | Malaysia |
| Carlos | Florez Zorrilla | Centro Medico Nacional 20 De Noviembre | Mexico City | Mexico |
| Sarnai | Erdene | Mongolian National University Of Medical Sciences | Ulaanbaatar | Mongolia |
| Moniba | Korch | University Hospital Mohammed Vi | Marrakech | Morocco |
| Iltimass | Gouazar | Faculté De Médecine Et De Pharmacie De Marrakech University Hospital Center Mohamed | Marrakesh | Morocco |
| Badr | Serji | Oncology Hospital Hasssan Ii. Faculty Of Medicine And Pharmacy Mohammed Ist University | Oujda | Morocco |
| Aziz | Zentar | Military Universitaire Hospital Med V Rabat Morocco | Rabat | Morocco |
| Amine | Benkabbou | National Institute Of Oncology, Mohammed V University In Rabat | Rabat | Morocco |
| Reda | Elhassouni | National Institute Of Oncology, Mohammed V University In Rabat | Rabat | Morocco |
| Sabrillah | Echiguer | National Institute Of Oncology, Mohammed V University In Rabat | Rabat | Morocco |
| Mohammed Anass | Majbar | National Institute Of Oncology, Mohammed V University In Rabat | Rabat | Morocco |
| Abdulrashid Pueya | Nashidengo | Windhoek Central Academic Hospital/University Of Namibia School Of Medicine | Windhoek | Namibia |
| Paleswan Joshi | Lakhey | Tribhuvan University Teaching Hospital, Institute Of Medicine | Kathmandu | Nepal |
| John | Windsor | Auckland City Hospital | Auckland | New Zealand |
| Michael Jen Jie | Chu | Auckland City Hospital | Auckland | New Zealand |
| Peter | Johnston | Auckland City Hospital | Auckland | New Zealand |
| Vanshay | Bindra | Waitemata District Health Board | Auckland | New Zealand |
| Andrea | Cross | Christchurch Hospital | Christchurch | New Zealand |
| Ashok | Gunawardene | Waikato Hospital | Hamilton | New Zealand |
| Fraser | Welsh | Waikato Hospital | Hamilton | New Zealand |
| Olusegun | Alatise | Obafemi Awolowo University | Ile Ife | Nigeria |
| Jibran | Abbasy | Aga Khan University Hospital | Karachi | Pakistan |
| Tayyab | Siddiqui | Aga Khan University Hospital | Karachi | Pakistan |
| Mohammad | Asghar | King Edward Medical University Mayo Hospital Lahore | Lahore | Pakistan |
| Mustafa | Abu Jayyab | Alshifaa Hospital | Gaza | Palestine, State of |
| Qusai | Zreqat | Al -Ahli Hospital-Hebron-Palestine | Hebron | Palestine, State of |
| Rawand | Titi | Alia Governmental Hospital -Hebron | Hebron | Palestine, State of |
| Fatima | Manasrah | Rafedia Government Hospital | Nablus | Palestine, State of |
| Ahlam | Hammoudeh | Palestine Medical Complex | Ramallah | Palestine, State of |
| Guillermo | Coayla | Hospital Nacional Edgardo Rebagliati Martins | Lima | Peru |
| Catherine | Teh | Makati Medical Center | Makati | Philippines (the) |
| Cenon | Alfonso | Ateneo School Of Medicine And Public Health, Dean | Pasig | Philippines (the) |
| Marta | Flisińska | Jurasz University Hospital | Bydgoszcz | Poland |
| Justyna | Rymarowicz | Universiy Hospital Krakow | Krakow | Poland |
| Marek | Sierzega | Jagiellonian University Medical College | Kraków | Poland |
| Wojciech | Ciesielski | Medical University Of Lodz | Lodz | Poland |
| Oliwia | Grząsiak | Medical University Of Lodz | Łódź | Poland |
| Patrycja | Szewczyk | Medical University Of Lodz | Łódź | Poland |
| Krzysztof | Szwedziak | Medical University Of Lodz | Łódź | Poland |
| Wojciech | Korcz | Medical University Of Warsaw | Warsaw | Poland |
| Oskar | Kornasiewicz | Oskar Kornasiewicz | Warsaw | Poland |
| Emanuel | Vigia | Hospital De Curry Cabral - Centro Hospitalar Universitário De Lisboa Central | Lisbon | Portugal |
| Raluca | Bievel Radulescu | Emergency Hospital Prof Dr. “Agrippa Ionescu” | Bucharest | Romania |
| Traian | Dumitrascu | Fundeni Clinical Institute | Bucharest | Romania |
| Mara | Mardare | Monza Hospital Bucharest | Bucharest | Romania |
| Octav | Ginghina | Saint John Emergency Hospital, Bucharest, Romania | Bucharest | Romania |
| Iulian | Brezean | Spitalul Clinic Dr. I. Cantacuzino | Bucharest | Romania |
| Sorin | Petrea | Spitalul Clinic Dr. I. Cantacuzino | Bucharest | Romania |
| Adrian | Bartos | ''Iuliu Hatieganu'' University Of Medicine And Pharmacy | Cluj-Napoca | Romania |
| Raluca | Bodea | Regional Institute Of Gastroenterology And Hepatology | Cluj-Napoca | Romania |
| Sergiu | Matei | University Hospital C.F.R. Cluj-Napoca, Romania | Cluj-Napoca | Romania |
| Ana-Maria | Musina | Regional Institute Of Oncology | Iasi | Romania |
| Natalia | Velenciuc | Regional Institute Of Oncology | Iasi | Romania |
| Alexander | Belyaev | Archangel Regional Clinical Hospital | Arkhangelsk | Russian Federation |
| Denis | Mizgirev | First Clinical City Hospital Named After E. E. Volosevich | Arkhangelsk | Russian Federation |
| Andrey | Litvin | Immanuel Kant Baltic Federal University, Regional Clinical Hospital, Kaliningrad | Kaliningrad | Russian Federation |
| Ivan | Semenenko | I.M. Sechenov First Moscow State Medical University (Sechenovskiy University) | Moscow | Russian Federation |
| Arkady | Bedzhanyan | Petrovsky National Research Center Of Surgery | Moscow | Russian Federation |
| Nikolay | Bagmet | Petrovsky National Research Center Of Surgery | Moscow | Russian Federation |
| Ayrat | Kaldarov | Vishnevsky Center Of Surgery | Moscow | Russian Federation |
| Denis | Kuchin | Privolzhsky District Medical Center | Nizhny Novgorod | Russian Federation |
| Evgeniy | Drozdov | Siberian State Medical University | Tomsk | Russian Federation |
| Mahir | Gachabayov | Vladimir City Emergency Hospital | Vladimir | Russian Federation |
| Mohammed | Alharthi | King Abdulaziz University Hospital | Jeddah | Saudi Arabia |
| Aleksandar | Bogdanovic | University Clinical Center of Serbia | Belgrade | Serbia |
| Daniel | Galun | University Clinical Center of Serbia | Belgrade | Serbia |
| Stefan | Kmezic | University Clinical Center of Serbia | Belgrade | Serbia |
| Dragana | Arbutina | Kbc Zvezdara Klinika Za Hirurgiju "Nikola Spasić" | Belgrade | Serbia |
| Mihailo | Bezmarevic | Military Medical Academy | Belgrade | Serbia |
| Jovan | Juloski | Zvezdara University Medical Center | Belgrade | Serbia |
| Mladjan | Protic | Oncology Institute Of Vojvodina | Novi Sad | Serbia |
| Brian | Goh | Singapore General Hospital | Singapore | Singapore |
| Blaž | Trotovšek | University Medical Centre Ljubljana | Ljubljana | Slovenia |
| Emil | Loots | Entabeni Hospital | Durban | South Africa |
| Jones | Omoshoro-Jones | Chris Hani Baragwanath Academic Hospital/University Of The Witwatersrand | Johannesburg | South Africa |
| Manuel | Marcello | Hospital Universitario Fundación Alcorcón | Alcorcón-Madrid | Spain |
| Jose | Ramia | Hospital General Universitario de Alicante Dr. Balmis | Alicante | Spain |
| Maria Del Mar | Rico-Morales | Hospital Universitario Torrecárdenas | Almería | Spain |
| Fabio | Ausania | Hospital Clinic, IDIBAPS, University of Barcelona | Barcelona | Spain |
| Ana Belen | Martin Arnau | Hospital de la Santa creu i Sant Pau | Barcelona | Spain |
| Benedetto | Ielpo | Hospital Del Mar | Barcelona | Spain |
| Esther Pilar | Santos | Hospital General Universitario De Ciudad Real | Ciudad Real | Spain |
| Patricia | Ruiz | Cruces University Hospital | Cruces (Bilbao) | Spain |
| Laia | Falgueras | Hospital Universitari De Girona Dr. Josep Trueta | Girona | Spain |
| Farah | Al Shwely | Hospital University Guadalajara | Guadalajara | Spain |
| Juli | Busquets | Bellvitge University Hospital | Barcelona | Spain |
| Iago | Justo | "12 De Octubre" University Hospital | Madrid | Spain |
| Jana | Dziakova | Hospital Clinico San Carlos | Madrid | Spain |
| Marcello | Di Martino | Hospital Universitario La Princesa | Madrid | Spain |
| Miguel Ángel | Suárez-Muñoz | University Hospital Virgen de la Victoria | Málaga | Spain |
| Lorena | Solar García | Hospital Universitarios Central de Asturias | Oviedo | Spain |
| Juan Jose | Segura-Sampedro | Hospital Son Espases | Palma De Mallorca | Spain |
| Fernando | Rotellar | Clinica Universidad De Navarra | Pamplona | Spain |
| Luis | Muñoz-Bellvis | Hospital Universitario De Salamanca. Universidad De Salamanca | Salamanca | Spain |
| Valle | Vera | Hospital Quiron Salud Infanta Luisa | Sevilla | Spain |
| María | García Domingo | Hospital Universitari Mutua Terrassa | Terrassa | Spain |
| Carlos | Domingo-Del Pozo | Hospital Universitario Dr. Peset | Valencia | Spain |
| Cristina | Ballester Ibáñez | Hospital Universitario La Fe | Valencia | Spain |
| Dimitri | Dorcaratto | Hospital Clínico Universitario de Valencia | València | Spain |
| Mario | Rodriguez-Lopez | Hospital Clínico Universitario de Valladolid | Valladolid | Spain |
| Alejandro | Serrablo | Miguel Servet University Hospital | Zaragoza | Spain |
| Hytham | Hamid | Ibn Sina Hospital | Khartoum | Sudan |
| Abdelfatah | Abdelmageed | Omdurman Islamic University | Khartoum | Sudan |
| Per | Sandström | University Hospital Of Linkoping | Linköping | Sweden |
| Linda | Lundgren | University Hospital Of Linkoping | Linköping | Sweden |
| Bobby | Tingstedt | Lund University | Lund | Sweden |
| Caroline | Williamsson | Lund University | Lund | Sweden |
| Bodil | Andersson | Lund University | Lund | Sweden |
| Ernesto | Sparrelid | Karolinska Institutet | Stockholm | Sweden |
| Christoph | Kuemmerli | Clarunis, St. Clara Hospital And University Hospital. | Basel | Switzerland |
| Beat | Moeckli | Hôpitaux Universitaire de Genève | Geneva | Switzerland |
| Alessandra | Cristaudi | Ospedale Regionale Di Lugano | Lugano | Switzerland |
| Fariba | Abbassi | Kantonsspital St.Gallen | St. Gallen | Switzerland |
| Jan | Schmidt | Hirslanden Clinics Zurich | Zurich | Switzerland |
| Stefan | Gutknecht | Triemli Hospital Zurich | Zurich | Switzerland |
| Jan Philipp | Jonas | University Hospital Zurich | Zurich | Switzerland |
| Mais | Alhashemi | University Of Aleppo | Aleppo | Syrian Arab Republic |
| Zain | Douba | University Of Aleppo | Aleppo | Syrian Arab Republic |
| Ahmad | Kayali | University Of Aleppo | Aleppo | Syrian Arab Republic |
| Oula | Azizeh | University Of Aleppo | Aleppo | Syrian Arab Republic |
| Omar | Al-Abed | University Of Aleppo | Aleppo | Syrian Arab Republic |
| Aya | Abdulmonem | Ain Shams University Hospitals | Cairo | Syrian Arab Republic |
| Ahmad | Alhouri | Al Assad University Hospital | Damascus | Syrian Arab Republic |
| Hasan | Al Houri | Al Assad University Hospital | Damascus | Syrian Arab Republic |
| Alnour | Suliman | Alasad Hospital | Damascus | Syrian Arab Republic |
| Bashar | Haj Hassan | Tishreen University Hospital | Lattakia | Syrian Arab Republic |
| Alaa | Hamdan | Tishreen University Hospital | Lattakia | Syrian Arab Republic |
| Ming-Chin | Yu | New Taipei Municipal Tucheng Hospital, Chang Gung Medical Foundation | New Taipei City | Taiwan (Province China) |
| Hanen | Bouaziz | Salah Azaiez Institut | Ariana | Tunisia |
| Ali | Kchaou | Habib Bourguiba University Hospital | Sfax | Tunisia |
| Ammar | Houssem | Sahloul Hospital | Sousse | Tunisia |
| Mesut | Tez | Ankara Numune Hospital | Ankara | Turkey |
| Mustafa | Kerem | Gazi University | Ankara | Turkey |
| Hüseyin | Bayhan | Gazi University | Ankara | Turkey |
| Ulaş | Aday | Dicle University School Of Medicine | Diyarbakır | Turkey |
| Alpen | Gumusoglu | Bakırkoy Dr. Sadi Konuk Research And Training Hospital | Istanbul | Turkey |
| Husnu | Aydin | Bakırkoy Dr. Sadi Konuk Research And Training Hospital | Istanbul | Turkey |
| Fatema | Hanefa | Istanbul University Faculty Of Medicine (Çapa) | Istanbul | Turkey |
| Ali Emre | Atici | Marmara University | Istanbul | Turkey |
| Hanife | Ulgur | Umraniye Training and Research Hospital | Istanbul | Turkey |
| Ismail | Sert | Egepol Surgery Hospital | Izmir | Turkey |
| Semra | Demirli Atici | University Of Health Sciences Tepecik Training And Research Hospital | Izmir | Turkey |
| Elif | Colak | Samsun Training And Research Hospital | Samsun | Turkey |
| Denys | Skoryi | Kharkiv Regional Center Of Oncology | Kharkiv | Ukraine |
| Kostiantyn | Kopchak | National Cancer Institute | Kyiv | Ukraine |
| Oleksandr | Kvasivka | National Cancer Institute | Kyiv | Ukraine |
| Valeriia | Sumarokova | National Cancer Institute | Kyiv | Ukraine |
| James | Skipworth | University Hospitals Bristol Nhs Trust | Bristol | United Kingdom |
| William | Lim | University Of Glasgow | Glasgow | United Kingdom |
| Ahmed | Mohamed | Royal Free London NHS Foundation Trust | London | United Kingdom |
| Pascale | Tinguely | Royal Free London NHS Foundation Trust | London | United Kingdom |
| Hemant | Kocher | The London Clinic | London | United Kingdom |
| Amjad | Khalil | Wellington Hospital, HCA | London | United Kingdom |
| Nicola | de' Liguori Carino | Manchester Royal Infirmary, University Of Manchester | Manchester | United Kingdom |
| Fahed | Gareb | Queen Elizabith Queen Mother Hospital | Margate | United Kingdom |
| Pandanaboyana | Sanjay | Freeman Hospital | Newcastle | United Kingdom |
| Krishnakumure | Patel | Nottingham University Hospitals | Nottingham | United Kingdom |
| Carlo | Ceresa | Oxford University Hospitals Nhs Foundation Trust | Oxford | United Kingdom |
| Thomas | Russell | University Hospitals Plymouth Nhs Trust | Plymouth | United Kingdom |
| Matt | Mortimer | Morristown Hospital | Swansea | United Kingdom |
| Megan | Sulciner | Brigham And Women'S Hospital | Boston | United States of America |
| Thomas | Clancy | Brigham And Women'S Hospital | Boston | United States of America |
| Martina | Nebbia | Massachusetts General Hospital | Boston | United States of America |
| Rachel | Thompson | Baylor University Medical Center | Dallas | United States of America |
| Dimitrios | Moris | Duke University Medical Center | Durham | United States of America |
| Jennifer | Gnerlich | Inova Schar Cancer Institute | Fairfax | United States of America |
| Richard | Spencer-Cole | University Of Arkansas For Medical Sciences | Little Rock | United States of America |
| Derek | Krinock | University Of Arkansas For Medical Sciences | Little Rock | United States of America |
| Tamara | Osborn | University Of Arkansas For Medical Sciences | Little Rock | United States of America |
| Hailey | Hardgrave | University Of Arkansas For Medical Sciences | Little Rock | United States of America |
| Joe | Nigh | University Of Arkansas For Medical Sciences | Little Rock | United States of America |
| Beth | Schrope | Columbia University | New York | United States of America |
| Ryan | Lamm | Thomas Jefferson University Hospital | Philadelphia | United States of America |
| Harish | Lavu | Thomas Jefferson University Hospital | Philadelphia | United States of America |
| Wilbur | Bowne | Thomas Jefferson University Hospital | Philadelphia | United States of America |
| Jonathan | Sham | University Of Washington | Seattle | United States of America |
| Paulo | Martins | University Of Massachusetts | Worcester | United States of America |
| Aisha | Albar | Sana'a University, Al-Thawra Modern General Hospital | Sana'A | Yemen |
| Fatima | Al-Eryani | Sana'a University, Al-Thawra Modern General Hospital | Sana'A | Yemen |
| Hamza | Al-Naggar | Sana'a University, Al-Thawra Modern General Hospital | Sana'A | Yemen |
| Rafat | Al-Saban | Sana'a University, Al-Thawra Modern General Hospital | Sana'A | Yemen |
